# Cardiac Roles of Serotonin (5-HT) and 5-HT-Receptors in Health and Disease

**DOI:** 10.3390/ijms24054765

**Published:** 2023-03-01

**Authors:** Joachim Neumann, Britt Hofmann, Stefan Dhein, Ulrich Gergs

**Affiliations:** 1 Institut für Pharmakologie und Toxikologie, Medizinische Fakultät, Martin-Luther-Universität Halle-Wittenberg, D-06097 Halle, Germany; 2 Cardiac Surgery, Medizinische Fakultät, Martin-Luther-Universität Halle-Wittenberg, D-06097 Halle, Germany; 3 Institut für Pharmakologie und Toxikologie, Medizinische Fakultät, Universität Leipzig, D-04109 Leipzig, Germany

**Keywords:** serotonin, 5-HT_4_-serotonin receptors, human atrium, human ventricle, transgenic mouse

## Abstract

Serotonin acts solely via 5-HT_4_-receptors to control human cardiac contractile function. The effects of serotonin via 5-HT_4_-receptors lead to positive inotropic and chronotropic effects, as well as arrhythmias, in the human heart. In addition, 5-HT_4_-receptors may play a role in sepsis, ischaemia, and reperfusion. These presumptive effects of 5-HT_4_-receptors are the focus of the present review. We also discuss the formation and inactivation of serotonin in the body, namely, in the heart. We identify cardiovascular diseases where serotonin might play a causative or additional role. We address the mechanisms which 5-HT_4_-receptors can use for cardiac signal transduction and their possible roles in cardiac diseases. We define areas where further research in this regard should be directed in the future, and identify animal models that might be generated to this end. Finally, we discuss in what regard 5-HT_4_-receptor agonists or antagonists might be useful drugs that could enter clinical practice. Serotonin has been the target of many studies for decades; thus, we found it timely to summarise our current knowledge here.

## 1. A Brief History of Serotonin and an Introduction to the Field

Serotonin can act via 5-HT_4_-receptors to control human cardiac contractile function. Therefore, it seems reasonable to put emphasis on these receptors and mention other serotonin receptors in the heart only briefly. They might be the subject to further reviews as soon as their role in the heart turns out to lead to drug targets. The effects of serotonin via 5-HT_4_-receptors lead to positive inotropic and chronotropic effects, as well as arrhythmias, in the human heart. In addition, 5-HT_4_-receptors may play a role in sepsis, ischaemia, and reperfusion. These presumptive effects of 5-HT_4_-receptors will play a major role in the present review. As a first step, we will go back some time in history.

Vittorio Erspamer from Italy derived extracts in organic solvents using intestinal preparations from several species (mainly rabbits). He studied the effects of these extracts on blood pressure or the contraction of isolated vessels from many species for approximately two decades. He called the active blood-pressure-raising agent “enteramine” because it came from the gastrointestinal tract. In 1952, his group discovered that enteramine was chemically 5-hydroxytryptamine (5-HT) [1]. The name enteramine is now no longer associated with 5-HT, but reminds one that large amounts of 5-HT are produced by intestinal cells of many species, including humans. Currently, the name serotonin is used for 5-HT. Serotonin was named as such when the group led by Irvine H. Page in the USA (Cleveland, Ohio) was looking for a cause of hypertension in patients. They screened blood from hypertensive patients and looked for chemical compounds that exhibited a lower concentration in sera from normotensive control persons. Using this strategy, they noticed a compound they called serotonin in extracts (from the serum of hypertensive patients that cause vasoconstriction in vitro) and showed that their serotonin was chemically identical with synthesised 5-HT [2]. Today, serotonin is usually thought of in connection with the brain. However, in 1953, 5-HT was found in the brain (review in [3]).

5-HT is phylogenetically a very old mediator and perhaps, therefore, involved in many physiological processes in the human body [4]: 5-HT is involved in organogenesis [5,6]; in the brain, 5-HT contributes to learning, feeding, sleeping, memory, mood, breathing [7,8], peripheral lung function [9], and aggression (reviewed in [10,11]). 5-HT might be involved in brain diseases such as schizophrenia, depression, compulsive/obsessive disorders, drug dependence, alcoholism, and autism (reviewed in [12]). Early on, a function of 5-HT in the gastrointestinal tract was noted: 5-HT typically increases the contraction of the intestine (reviewed in [13]). 5-HT has also been suggested to be involved in Morbus Crohn, Colitis ulcerosa, coeliac disease, and diverticulitis [13].

**Figure 1:**
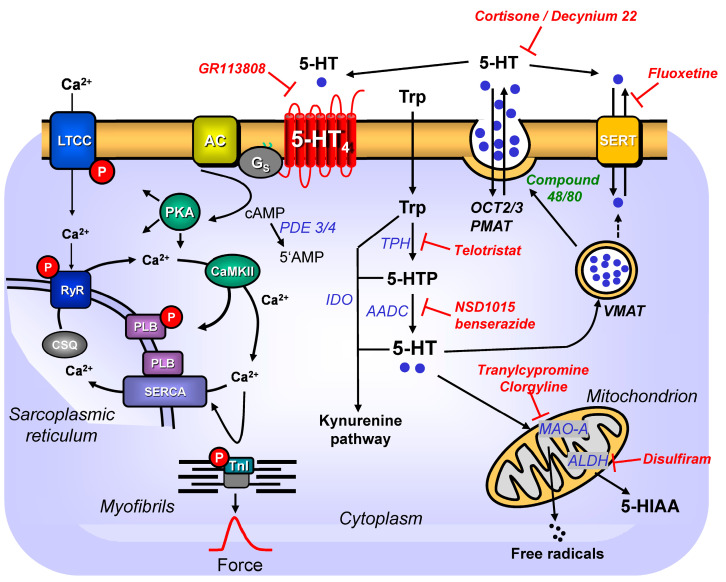
Putative metabolism of serotonin in the heart. Activation of 5-HT_4_-receptors (5-HT_4_) leads to stimulatory guanosine triphosphate proteins (Gs) and increases the activity of adenylyl cyclases (AC5/6). This stimulation is antagonised by, e.g., GR 113808. More 3′,5′ cyclic adenosine monophosphate (cAMP) is formed that activates the cAMP-dependent protein kinases (PKA). This leads to the phosphorylation and activation of L-type calcium channels (LTCC) in the sarcolemma, of the ryanodine receptors (RYR), phospholamban (PLB, on amino acid serine 16) and the inhibitory subunit of troponin (TnI). Calcium ions enter the cytosol from the LTCC or via the RYR. In cytosol, calcium ions bind to myofilaments, which enhances force generation. Calcium ions also activate a calcium and calmodulin protein kinase (CaMK) that likewise phosphorylates RYR and PLB (on amino acid threonine 17). The generated cAMP in the heart is mainly degraded by phosphodiesterase (PDE) 3 or 4. Calcium ions in the sarcoplasmic reticulum (SR) are stored by binding to calsequestrin (CSQ). Calcium is pumped back from the cytosol by SR-Ca-ATPase (SERCA). The activity of SERCA is impaired by PLB and enhanced by phosphorylated PLB. Tryptophan (Trp) enters the cardiomyocyte where it is hydroxylated by tryptophan hydroxylase 1 (inhibitable by, e.g., telotristat) to 5-hydroxy-tryptophan (5-HTP). 5-HTP can then be decarboxylated by amino acid decarboxylase (AADC, inhibitable by NSD 1015 or benserazide) to serotonin (5-HT). Alternatively, tryptophan can be metabolised by indole amine dioxygenase (IDO) and enters the kynurenine pathway. 5-HT can be oxidated by mono amine oxidases (MAO-A, inhibitable by clorgyline or tranylcypromine) located in the outer membrane of mitochondria. Thereafter, the products can be further degraded by an alcohol dehydrogenase (inhibitable by disulfiram). 5-HT can be transported into storage vesicles via a vescular mono amine transporter (VMAT). 5-HT can exit the cell via serotonin transporter (SERT, inhibitable by fluoxetine) or via OCT or PMAT (inhibitable by cortisone or decynium 22).

5-HT hardly passes through the intact blood–brain barrier [12]. Thus, the separate production of 5-HT in the brain and peripheral organs must occur. One assumes that 95% of human 5-HT is present in the peripheral organs, and only 5% is present in the human brain [14]. It is further assumed that 90% of 5-HT found in the peripheral blood of humans is formed in enterochromaffin cells in the gut, and approximately 10% is formed in neurons of the gastrointestinal tract (reviewed in [13,15]). In the peripheral blood, more than 95% of 5-HT is present in platelets. In enterochromaffin cells, 5-HT is formed via the rate-limiting enzyme tryptophan hydroxylase 1. In contrast, in the serotonergic neurons of the gut (but also in serotonergic brain neurons), 5-HT is synthesised via an enzyme coded by a different, but functionally similar, gene from tryptophan hydroxylase 1, called tryptophan hydroxylase 2 (reviewed in [14,16]). Surprisingly, in the plasma of mice, where the genes for tryptophan hydroxylase 1 and tryptophan hydroxylase 2 were deleted, measurable levels of 5-HT were still detected (reviewed in [17]). This was interpreted as evidence that 5-HT can also be formed from phenylalanine hydroxylase, which can hydroxylate tryptophan but with low velocity (reviewed in [17]) (Figure 1).

As predicted from the structural formulae, phenylalanine derivatives have been synthesised that inhibit tryptophan hydroxylase activity because they are false substrates (reviewed in [17]). In recent years, newer compounds have been described that are more specific than older inhibitors and do not pass through the blood–brain barrier. In other words, in our case, they would only inhibit peripheral 5-HT formation in the heart (reviewed in [17]). In oncology, to treat carcinoid syndrome, tryptophan inhibitors (e.g., telotristat: Figure 1) have found approval from regulatory authorities (reviewed in [17]). It might be instructive to study cardiac function in such patients: one might predict a lower beating rate and lower incidences of arrhythmias. Experimental animal studies suggest that tryptophan inhibitors might be beneficial in treating pulmonary hypertension (reviewed in [17]).

Interestingly, 5-HT is transported via the vesicular monoamine transporter 1 (VMAT 1) into the storage vesicles of enterochromaffin cells [18]. 5-HT can spontaneously or, after mechanical or chemical irritation, leave enterochromaffin cells. Mechanical irritation can be the contraction of the gut [19]. Small molecules, such as fatty acids from gut bacteria (forming the gut microbiota), can elicit the release of 5-HT from enterochromaffin cells [20]. The high glucose content of food will also increase the 5-HT secretion of enterochromaffin cells [18]. The release of 5-HT occurs in the gut and plasma sides of enterochromaffin cells. On the plasma side, 5-HT is transported into platelets via (serotonin transporter) SERT (reviewed in [2,21]). This offers the interesting possibility that the gut microbiome may indirectly regulate 5-HT levels in platelets [20]. Thus, platelets contain the bulk (about 95%) of 5-HT that enters the heart capillaries via the coronary arteries [20]. Nevertheless, nearly all other cell constituents of the blood contain some 5-HT; for example, even immune cells might transport some 5-HT into the human heart. On the outer cell membranes of platelets, one finds 5-HT_2A_-receptors [22]. The activation of 5-HT_2A_-receptors by 5-HT is thought to amplify platelet aggregation [23].

The enzyme tryptophan hydroxylase (TPH, see next paragraph) oxidises the amino acid L-tryptophan. Tryptophan has been administered to animals or humans orally, which is sufficient to raise body concentrations of tryptophan [24]. Hence, peroral tryptophan, sometimes taken as a food supplement by the general population or athletes, might lead to higher serotonin concentrations in the brain and heart [25]. However, the direct effect of peroral tryptophan on 5-HT levels in human hearts has, to the best of our knowledge, never been reported. Any 5-HT produced in the intestine should enter the heart and be stored in platelets. This stored 5-HT can pass the membrane of platelets when they become activated platelets. The activation of platelets might occur when thrombi are formed in the heart. The formation of thrombi is often caused by cardiac arrhythmias, such as left or right atrial fibrillation [26]. Serotonin can also cause cardiac arrhythmias [27,28]. Hence, in an apparent vicious circle, 5-HT can induce arrhythmias, which can form thrombi, and these thrombi lead to the release of more 5-HT from platelets in the heart (reviewed in [28]).

Once 5-HT exits the thrombi, there are several ways it may alter cardiac function and induce arrhythmias. For example, 5-HT from platelets can easily pass the short distance to reach endothelial cells. On the surface of endothelial cells, 5-HT receptors can be activated, and thus usually lead to vasodilatation, which might be cardioprotective (reviewed in [28]). If these endothelial cells were lacking (for instance, after local injury or due to atherosclerosis), 5-HT might act on 5-HT-receptors on the outer surface of smooth muscle cells where stimulation of serotonin receptors (mainly the 5-HT_1_-receptor) induces vasoconstriction that might precipitate the formation of activated platelets, the release of 5-HT, and the formation of thrombi (reviewed in [28]). Furthermore, 5-HT originating from platelets might diffuse deeper into the tissue and activate 5-HT receptors in cardiomyocytes (reviewed in [28]). These 5-HT receptors on cardiomyocytes might alter ion channel function in the sarcolemma so that depolarisation of the cardiomyocyte occurs and arrhythmias start or are sustained (see below). Furthermore, the 5-HT-producing enzyme TPH1 was found in pulmonary endothelial cells and cardiomyocytes using immunohistology [29]. Hence, gut cells, and even heart cells, may produce serotonin, which might act in an autocrine or paracrine fashion.

Tryptophan hydroxylase 1 (TPH1, named so because it was the first isoform detected) is usually found in the body’s periphery. TPH2 is highly abundant in the central nervous system, but is also found in neuronal cells of the gut [30]. The complete deletion of TPH1 decreased the cardiac (adult mouse) concentration of 5-HT to approximately 10% that of wild-type levels. This might mean that considerable amounts of 5-HT are produced in the heart [30]. Some, but not all, TPH1-knockout mice develop heart failure [30], meaning that 5-HT is necessary for heart function. In Northern blots and reverse transcriptase polymerase chain reactions (RT-PCRs) using HL-1 cells (a rat-heart-derived tumour line) and neonatal rat heart cells or hamster adult hearts, several groups have detected TPH1 [31,32]). In contrast, TPH2 was not measurable even with RT-PCR in cardiac tissue [31,32,33,34]. Consistent with these RT-PCR data, Western blotting identified TPH1 in whole hearts from adult mice and rats. Fittingly, no TPH1 was detectable in TPH1-knockout mouse hearts [34]. However, the localisation of TPH1 is uncertain: in rat hearts, but not in mouse hearts, TPH1 is located in cardiac mast cells [34]. However, this might be antibody-dependent, because others found TPH1 in mouse ventricular heart cells and human atrial heart cells from surgical patients [29].

Amino acid decarboxylase (AADC) [35] was detected using RT-PCR only in neonatal rat cardiomyocytes [31]. AADC will decarboxylase 5-hydroxytryptophan, leading to a new molecule, namely, 5-HT. Somewhat surprisingly, others found AADC only in endothelial cells, but not in the cardiomyocytes of adult rat hearts and adult mouse hearts [34]. It remains to be studied whether this is due to age differences or technical difficulties in detecting AADC, which could be determined by conducting a time course study in rat hearts. Others could detect AADC in adult mouse cardiomyocytes and human right atria from adult patients [29]. Moreover, the addition of 5-hydroxytryptophan (the direct precursor of 5-HT) augmented 5-HT levels in cardiac mouse myocytes [36]. Interestingly, 5-hydroxytryptophan, albeit at higher concentrations than 5-HT, has functional consequences in the heart. More specifically, in electrically stimulated left atrial preparations of mice overexpressing 5-HT_4_-receptors selectively in the heart (5-HT_4_-TG), 5-hydroxy-tryptophan (5-HTP) exerted time- and concentration-dependent positive inotropic effects and increased the beating rate in the right atrial preparations [29].

More relevant is that 5-HTP augmented the force of contraction in isolated human atrial preparations [29]. The injection of 5-HTP into intact mice led to an increase in 5-HTP in the cardiac tissue of mice [29,34]. Fittingly, when benserazide was applied in living mice, the investigators found reduced concentrations of 5-HT in the heart [34]. Similarly, the application of 5-HPT in isolated buffer-perfused hearts led to a measurable increase in the cardiac content of 5-HT [34], and the effect was blocked by the injection of benserazide (an AADC inhibitor, used in the treatment of Parkinson’s disease). These authors concluded that 5-HTP, perhaps formed in platelets, might lead to higher concentrations of 5-HT in the heart [34]. In contrast, we suggest that 5-HT formation in the heart from 5-HTP may also contribute to their results.

The AADC gene has been genetically deleted in the kidney. This led to hypertension, because the blood pressure-lowering effects of dopamine were missing. Moreover, possibly due to lasting systemic hypertension, cardiac hypertrophy was noted [37]. Interestingly, cardiac-specific knockout was performed [38]. The cardiac genetics of AADC are puzzling. In the heart (but not in other tissues), AADC comes only from the parental lineage (human: [38], mouse: [39]. Moreover, knockout in mouse embryos of AADC had detrimental results, suggesting an essential role for AADC in the proper development of the foetal heart [38]. It would be interesting to study these mice with a deletion of AADC in more detail. One might suspect that 5-HT levels in the heart are diminished. Contractility to 5-HT is not expected to be altered in the deletion of AADC in the heart because contractility is not altered in wild-type mouse hearts, probably because the mouse 5-HT_4_-receptor is not functional [40].

## 2. Transport of 5-HT

Within cells, for instance, thrombocytes, 5-HT is either degraded via oxidation (as mentioned above) or transported intact using a protein called VMAT2 into vesicles, where 5-HT can be stored [41,42]. Other isoforms of VMAT are known. VMAT1 is found primarily in adrenal gland cells, whereas in the brain, as in platelets, VMAT2 is mainly used. VMAT1 and VMAT2 are also present in other non-neuronal cells, such as heart or ear saliva cells [43] or tubular cells in the kidney [44]. VMAT1 and VMAT2 protect 5-HT from degradation. However, following signals from cell surface receptors, these vesicles can translocate to the outer membranes. There, the vesicles will fuse with the outer cell membrane and empty their content, comprising 5-HT (but also many other small and large molecules) into the interstitium or platelets into the plasma. In this way, elevated local concentrations of 5-HT can occur. Such augmented levels of 5-HT are sufficiently elevated to serotonylate the proteins (see above).

5-HT can be produced within mouse and human cardiomyocytes; therefore, such intracellularly produced 5-HT could leave the cardiomyocyte. The uptake of 5-HT into non-neuronal cells is presumably mediated by transporter proteins such as organic cation transporter 1 (OCT1), OCT2, OCT3, and plasma membrane monoamine transporter (PMAT). PMAT, OCT2, and OCT3 have been histologically identified in mouse cardiomyocytes [29]. OCT2, OCT3, and PMAT can be found with specific antibodies in mouse or human cardiomyocytes [29] and by immunofluorescence (OCT1, OCT3) in the human heart [45].

## 3. Degradation of 5-HT

5-HT can be inactivated and oxidised by monoamine oxidase-A (MAO-A). In adult mouse cardiac myocytes, levels of 5-HT were highly elevated in the presence of tranylcypromine, which inhibits MAO-A and MAO-B [36] or in the presence of clorgyline inhibits MAO-A [36] but not by deprenyl, an MAO-B-inhibitor [36]. MAO is especially active in the gut, liver, and serotoninergic nerve cells. However, MAO-A and MAO-B were also histologically detected in mouse cardiomyocytes [29,46].

These enzymes are also functionally relevant for the metabolism of 5-HT in the mammalian heart because the positive inotropic effect of tranylcypromine occurred in wild type mice (WT) and 5-HT_4_-TG but was blocked in WT by propranolol but not in 5-HT_4_-TG [29]. This observation is consistent with the view that MAO can degrade 5-HT in the mouse heart. It is unclear from these data whether MAO-A or MAO-B are more relevant to degrading 5-HT because tranylcypromine is an irreversible inhibitor of both MAO-A and MAO-B [29,47]. However, MAO-A is more active in rat hearts than in MAO-B; in human hearts, MAO-A and MAO-B are both relevant [48]. The total activity of MAO was measured to be about a hundred-fold higher in the rat heart than in the wild-type mouse heart [49]. Likewise, MAO-B was more active than MAO-A in mouse hearts [50]. Thus, we would argue that the knockout of MAO-A in mice [51] is not the best model for studying the relative role of MAO-A and MAO-B in the human heart. MAO-A and MAO-B were regionally differently expressed in rat hearts [52].

Moreover, 5-HT is degraded by means of arylalkylamine-N-acetyltransferase (cardiac expression: [53]). After 5-HT is metabolised by MAO-A or MAO-B, 5-hydroxy-indole-acetaldehyde is formed. This latter molecule can be further broken down by dehydrogenases or alcohol dehydrogenase 2; eventually, 5-hydroxy-indole-acetic acid is formed, which exits the body [54,55]. In mice, 5-HT is thought to be mainly degraded by MAO-A, not MAO-B. When we reduced the action of MAO by applying tranylcypromine, we could potentiate the positive inotropic effect of 5-HT in atrial preparations of 5-HT_4_-TG [29]. Moreover, 5-HT is also metabolised by an indoleamine 2,3-dioxygenase (found in mouse cardiomyocytes [56]) to kynurenine (found in the murine heart: [57]). Indoleamine 2,3-dioxygenase can be induced in infectious diseases (cardiac viral myocarditis [56]) and knockout mice for this enzyme are available [56], which might help better understand the biological role of this enzyme for 5-HT metabolism.

The uptake of 5-HT into nerve cells is brought about by SERT [58]. SERT activity is reduced by typical antidepressant drugs, such as fluoxetine. The genetic deletion of SERT is accompanied by a decrease in 5-HT concentrations from 29 µM to 0.4 µM in whole blood, presumably via SERT into platelets. The EC_50_ value of 5-HT in the presence of cocaine for a positive inotropic effect is lower in human-isolated atrial preparations (39 nM) than in the absence of cocaine (230 nM: [59]). Thus, cocaine seems to inhibit the uptake into nerve cells via SERT, or 5-HT inhibits the uptake of 5-HT into cardiomyocytes by inhibiting SERT in cardiomyocytes. Indeed, SERT expression can be detected histologically in mouse cardiomyocytes [29]. SERT was also detected in the lung endothelial cells and smooth muscle cells [60], rat aorta [61], rat cardiac valves [62], dogs [63], human valvular tissue [33], and in the conduction system of mice and mouse cardiac endothelial cells [64,65,66].

Moreover, in foetal cardiomyocytes, SERT is seen in immunohistology [67]. SERT was further detected in the endocardium and endothelium of coronary arterial cells and capillaries, although SERT could not be detected in cardiomyocytes from adult mice [33]. Using different experimental conditions, we identified SERT in cardiomyocytes from adult mouse hearts and the human right atrium [29]. Functional evidence for the activity and, therefore, the presence of SERT was also reported; 5-HT, applied in the cell culture of adult rat ventricular myocytes, led to cellular hypertrophy, which was reduced by imipramine [68]. This may indicate that cardiomyocytes can take up 5-HT, and one could argue for the involvement of SERT in this process. The knockout of SERT in mice led to an approximately tenfold reduction in 5-HT levels in whole blood [33]. Adult mice with a global knockout of SERT developed cardiac dilatation and heart failure, possibly caused by ventricular and cardiac valve fibrosis. The effects are also present on 5-HT_1B_ receptor knockout mice, and hence are not 5-HT_1B_-receptor-mediated [33]. SERT acts as a reversible transporter during ischaemia in the presence of tyramine or amphetamines. Intracellular 5-HT could exit mouse cardiomyocytes [64]. Fluoxetine can shift the concentration–response curve for the positive inotropic effect of 5-HT to lower concentrations of 5-HT in the left atrium of mice overexpressing the 5-HT_4_-receptor [29]. This finding is consistent with the role of SERT in the heart.

## 4. Levels of 5-HT in the Heart

In adult mouse cardiomyocytes, the 5-HT level was approximately 2.9 pmol/mg protein [36]. Concentrations of 5-HT in isolated samples from human hearts (freshly frozen, after the autopsy, from the right atrium, from papillary muscles) were reported to range from 0.08 to 0.4 µg/g [69], recalculated as approximately 0.45 μM to 2.3 μM. Such differences might be due to platelet contamination (for very high values) or post-mortal degradation (for low levels). Assuming a homogenous distribution of 5-HT in isolated adult mouse cardiomyocytes, intracellular concentrations of 200 nM for 5-HT have been calculated [36]. These concentrations are well within the range of EC_50_ values for the 5-HT receptors, such as those responsible for inotropy in some mammalian species, including humans [21].

5-HT has been identified in samples from hamster hearts [69], samples from mouse hearts [30], and dog hearts [70], but most importantly, in samples from human hearts [69]. Against these studies, one could argue that the measurable 5-HT concentrations in these samples might simply originate from contaminant platelets (or mast cells) still present in the heart slices used for biochemical measurements of 5-HT. Alternatively, one could argue that 5-HT was present in non-cardiomyocytes, such as endothelial cells, smooth muscle cells, or fibroblasts, in these samples. In other words, one might question whether the 5-HT measured in these studies was present in cardiomyocytes. However, in later studies, 5-HT was found in cultured rat neonatal cardiomyocytes [31]), rat primary cardiac myofibroblasts [71], and freshly isolated adult mouse cardiomyocytes [36]. Hence, one can now be reasonably confident that 5-HT is present in mammalian hearts and cardiomyocytes.

## 5. 5-HT_4_-Receptor-Independent Actions of 5-HT

Interestingly, 5-HT can exert, in a receptor-independent fashion, intracellular effects when it is oxidised in mitochondria (e.g., in mouse cardiomyocytes). This oxidation of 5-HT leads to the generation of potentially nefarious free radicals. Through this oxidation, 5-HT as a molecule can directly cause apoptosis and necrosis [14,72]. There is a theory that in the Earth’s early evolution, when toxic oxygen levels were reached in the atmosphere, living cells started to use serotonin as an antioxidant to protect against the high partial pressure of oxygen. Hence, one could argue that the direct action of serotonin on mitochondria is a remnant of evolution.

There is another way, besides oxidation, in which 5-HT can act independently of a receptor on the cell surface. Via its primary amino group, 5-HT may covalently link proteins on the surface or in the cell. This covalent modification can change the function of these proteins. This covalent modification may be called serotonylation. This serotonylation is catalysed by a family of enzymes termed transglutaminases. For example, transglutaminase can connect 5-HT firmly to fibrinogen, small G-proteins, and several other proteins in platelets (reviewed in [73,74]). One can assume that serotonylation of these proteins occurs wherever they exist, i.e., in cardiomyocytes. Interestingly, in serotonylation, even histones can occur and alter their function. Thus, serotonin may alter cardiac gene transcription directly without the involvement of a receptor. We speculate that this is a very old evolutionarily conserved action of serotonin. One would predict that histones in cardiomyocytes can also be serotonylated [73]. This might be another way that 5-HT within the cardiomyocyte might alter signal transduction and gene expression in addition to acting via the classical transcription factor called cAMP-response element binding protein (CREB). However, this topic requires more research efforts.

## 6. Cardiac Contractile Effects of Serotonin with a Focus on 5-HT_4_-Receptors

A positive inotropic effect of 5-HT has been observed in the hearts of many mammalian species (Table 1). More specifically, positive inotropic effects were noted in cardiac preparations from cats, guinea pigs, dogs, pigs, and rats (Table 1) [75,76,77,78]. The positive inotropic effect in cats and other species was explained by the release of endogenous noradrenaline [79]. The positive inotropic effect in the same species might be region-dependent. For instance, in rats, a positive inotropic effect in the left atrium, but not in the papillary muscle, could be seen [80]. Similarly, in human atrial preparations, but not ventricular preparations, 5-HT led to a positive inotropic effect [81,82,83].

To summarise these data: 5-HT_4_-receptors mediate the cardiac effects of 5-HT in human cardiomyocytes. The only non-transgenic animal model for the 5-HT_4_-receptor is the pig heart [141]. There is the possibility of inducing stressors such as day-long ischaemia (in living animals) or hypertension in rats [127,128]. Thereafter, in addition to their physiological 5-HT_2A_-receptors, these rats suddenly show increased expression of the 5-HT_4_-receptor signal through 5-HT_4_-receptors [127]. Similarly, the transfection of adult rat cardiomyocytes with a virus coding for 5-HT_4_-receptors forced the rat cardiomyocytes to signal through this new 5-HT_4_-receptor [142]. Finally, one has generated and repeatedly studied transgenic mice that overexpress human 5-HT_4_-receptors [40]. All these animal models of human 5-HT_4_-receptors have their limitations. We aim to point this out clearly in this review. The “gold standard” is studies in human cardiac preparations. It must be pointed out that human tissue also comes with a burden. Patients have diseased hearts; a long anamnesis of pharmacotherapy and ethical concerns usually limit human studies. Typically, 5-HT does not increase the force of contraction in the human ventricle. However, a positive inotropic effect was sometimes reported in the presence of cyclic adenosine 3′,5′ monophosphate (cAMP)-increasing agents. For instance, phosphodiesterase (PDE) inhibitors or prostaglandin E1 revealed a positive inotropic effect in isolated muscle strips from the human ventricle [143,144].

## 7. Role of Phosphodiesterases (PDEs, Figure 2) and 5-HT_4_-Receptors

In ventricular preparations from patients with severe heart failure, 5-HT could slightly increase the force of contraction [143]. The positive inotropic effect was greatly augmented by the unselective PDE inhibitor 3-isobutyl-1-methylxanthine (IBMX) [84,143]. Fittingly, only in the presence of IBMX in ventricular preparations of pigs was a positive inotropic effect to 5-HT reported [112]. The role of individual PDEs has also been studied in left ventricular muscle samples from human hearts, where 5-HT exerted a very small positive inotropic effect (around 5%; [143]). This effect was significantly enhanced if the samples were pre-stimulated with 1 µM cilostamide (a selective PDE 3 inhibitor [143]). The effect of cilostamide could not be amplified by erythro-9-amino-β-hexyl-α-methyl-9H-purine-9-ethanol (EHNA), a PDE 2 inhibitor, but was augmented nominally by rolipram, a PDE 4 inhibitor [143]. The interpretation was that cAMP generation by 5-HT in the human heart is attenuated by the combined action of PDE 3 and 4 in the human ventricle [143].

**Figure 2:**
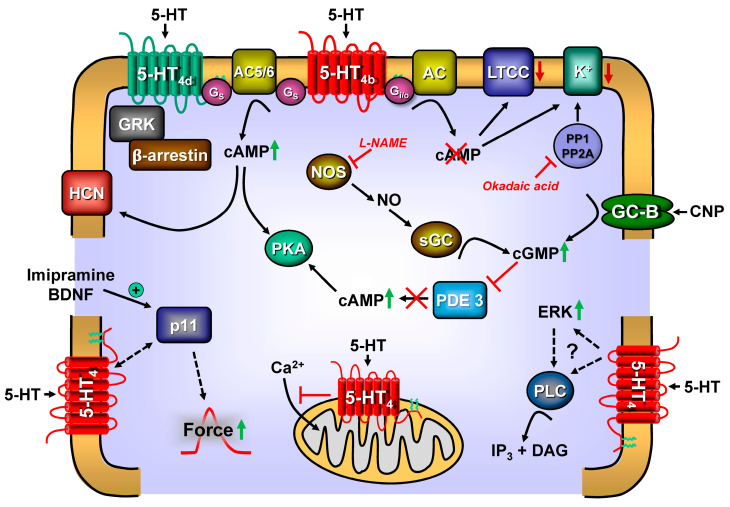
Synopsis of putative signal transduction of 5-HT_4_-receptors in the heart. For general information and abbreviations, see Figure 1. 5-HT_4a_-receptors couples only via stimulatory G-proteins (G_s_). 5-HT_4b_-receptors couple via both Gs and inhibitory G-proteins (G_i_). The 5-HT_4_-receptors can signal via guanosine triphosphate–protein couple receptor kinases (GRK). The 5-HT_4_-receptors can be phosphorylated and inactivated via GRK. The cAMP can bind and activate a hyperpolarisation-activated cation-channel (HCN), inducing tachycardia in the sinus node of the heart. Moreover, imipramine or BDNF treatment increase the expression of PIN1. Augmented levels of PIN1 can facilitate the coupling of 5-HT_4_-receptors to force generation. The coupling of 5-HT_4_-receptors to potassium channels (K^+^) is activated by PKA and reversed by the activity of serine/threonine protein phosphatases (PP1/PP2A inhibitable by okadaic acid). Via guanylyl cyclase-B (GC-B) receptor, the C-type natriuretic protein (CNP) can raise 3′,5′ cyclic guanosine monophosphate (cGMP) levels in cells. This cGMP can inhibit phosphodiesterase (PDE) 3 (III). If PDE 3 is inhibited, the cAMP-levels increase because degradation of cAMP is protracted. 5-HT_4_-receptors are also found on mitochondria, where they impair calcium ion influx. Nitric oxide (NO) synthase (NOS) can be phosphorylated and activated by PKA. NOS (inhibitable by L-NAME) can generate NO, which leads to the activation of a soluble guanylyl cyclase (GC), leading to the generation of more cGMP. Moreover, 5-HT_4_-receptors can activate extracellular-regulated-kinase (ERK) and increase the activity of protein kinase C via the previous activation of phospholipase C (PLC). This is achieved because PLC leads to the formation of inositol-trisphosphate (IP3) and diacylglycerol (DG). DG can activate protein kinase C.

Moreover, there is a hierarchy of PDEs: PDE 4 inhibition is only of functional relevance for 5-HT-induced positive inotropy if PDE 3 is also inhibited. Thus, PDE 4 alone is not relevant in the human ventricle, at least concerning 5-HT-mediated inotropy [143,145]. Similar data on the role of PDEs in human atrial tissues have been presented. However, the interpretation was different; there is a pronounced positive inotropic effect of 5-HT alone in human atrial strips [59] that could be amplified when PDEs are inhibited [146,147]. Interestingly, in permanent atrial fibrillation, the ability of 5-HT to increase the force of contraction in isolated atrial trabeculae is attenuated compared with control samples (sinus rhythm: [104]). In human atrial preparations in sinus rhythm, cilostamide alone, but not rolipram alone, increases the basal force of contraction [105]. The potency of 5-HT to increase force of contraction was enhanced by cilostamide (300 nM) alone, but not rolipram (1 µM) alone.

In contrast, the combination of cilostamide (300 nM) and rolipram (1 µM) shifted the concentration–response curve of 5-HT to the left of the effect of cilostamide (300 nM) alone [105], similar to the findings in human ventricular preparations [143]. While cilostamide (300 nM) but not rolipram (1 µM) increased the current through the L-type Ca^2+^ channels in atrial cardiomyocytes from patients in sinus rhythm, neither cilostamide nor rolipram enhanced the effect of 10 µM 5-HT on the current through the L-type Ca^2+^ channels any further [105]. These data confirm, in a sense, the contractile studies: cilostamide increased force and current. In contrast, rolipram increased neither force nor current, emphasising the different roles of PDE 3 and PDE 4 in the human heart [105].

Moreover, rolipram (1 μM) alone, cilostamide alone (300 nM), or their combination failed to increase the force of contraction in human atrial preparations in vitro, which is somewhat unexpected [119]. However, rolipram and cilostamide combined (but not when given alone) reduced the fading (reduction in force of time) of a single concentration of 5-HT (1 µM) [119]. Likewise, in failing rat ventricles, a positive inotropic effect via 5-HT_4_-receptors could be augmented by cilostamide alone, but not by EHNA or rolipram alone [143]. As with the human ventricle, the positive inotropic effect of 5-HT can only be noticed by applying rolipram, but not EHNA together with 5-HT [143]. This might mean a similar role for PDE 2, 3, and 4 in failing rat and human hearts, and might mean that, in this respect, the rat is a valuable model for failing human hearts. Also of interest is the non-failing (control) rat ventricle, which did not react to 5-HT alone [80]. Rolipram and cilostamide together revealed a positive inotropic effect of 5-HT. However, the authors did not report how cilostamide, rolipram, or EHNA alone could (or failed to) augment the positive inotropic effect of 5-HT in the control rat ventricle [143]. Such data would tell us whether, at least in the rat ventricle, selective upregulation or downregulation of the function of the isoenzymes of PDE might occur, altering the function of 5-HT. This might explain whether PDE can form boundaries or even compartments in this model of heart failure.

## 8. CNP (Figure 2) and 5-HT_4_-Receptors

C-type natriuretic peptide (CNP) stimulates B-type natriuretic peptide receptors, which finally leads to increased cellular levels of cyclic guanosine 3′,5′-monophosphate (cGMP). cGMP is degraded in the heart by at least PDE 3. This cGMP can inhibit PDE 3 activity, leading to increased levels of cAMP in cardiac cells. When one pre-treated ventricular preparations from rats with heart failure (induced by occluding coronary flow) and therefore functionally active 5-HT_4_-receptors, with 300 nM CNP, one noted that the efficacy of 5-HT to increase the force of contraction was increased [145]. If, in addition to 300 nM CNP, one was also pre-treated with 10 µM rolipram, the efficacy of 5-HT to increase force was elevated further by rolipram, which was accompanied by an increase in the potency of 5-HT to increase the force of contraction [145]. In contrast, CNP, combined with cilostamide, failed to increase the efficacy of 5-HT. This was regarded as evidence that the effect of CNP on 5-HT was mediated by the inhibition of PDE resulting from the formation of cGMP in the cells [145]. These interpretations were supported by the fact that L-N-nitro-arginine-methyl-ester (L-NAME), an inhibitor of nitric oxide activity known to inhibit the generation of cGMP by this mechanism, reduced the efficacy of 5-HT to increase the force of contraction [145]. It would be interesting to perform a confirmatory experiment: one could increase cellular cGMP by inhibiting PDE 5, which is a cGMP-specific PDE. In addition, cGMP-dependent protein kinase could be activated by administering 8-bromo-cGMP to muscle strips. One would predict that, under these conditions, the efficacy of 5-HT would increase.

Moreover, it would also be interesting to test [145] human atrial and human ventricular preparations under these conditions from non-failing and failing hearts because, up to now, only failing rat hearts have been studied [145]. From a mechanical point of view, it would be helpful to perform such experiments [145] in cardiomyocytes (preferably from humans). One could argue that the previously described experiments [145] might be a combination of CNP acting on cardiac endothelial or smooth muscle cells. In these cells, nitric oxide synthases (NOS) form nitric oxide (NO). NO can quickly diffuse into cardiomyocytes and activate guanylate cyclase to generate cGMP within these cardiomyocytes. To prevent the indirect effects of CNP or L-NAME, it would be informative to repeat these studies with cardiomyocytes (e.g., from a failing rat ventricle). At least CNP has been shown to increase cGMP and cAMP levels in cardiomyocytes from failing and non-failing rat hearts [148]. CNP increased the local concentrations of cGMP in the vicinity of phospholamban and the inhibitory subunit of troponin [149]. CNP also increased the phosphorylation state of PLB at serine 16 and the troponin inhibitor at serine 22/23 via the activation of a cGMP-dependent protein kinase [149,150,151]. However, the role of 5-HT has not been studied [148].

## 9. Desensitisation (Figure 2) of 5-HT_4_-Receptors

At high concentrations of 5-HT for prolonged times in an organ bath, a second attenuated positive inotropic effect of 5-HT was noted, which was alternatively explained as desensitisation by activating phosphodiesterases. Homologous desensitisation in the isolated atrium (also in the left ventricle of the living animal) can clearly be seen for the positive inotropic effect of the 5-HT in 5-HT_4_-receptor-overexpressing mice [152], as in isolated human cardiac preparations [28]. In vitro, high concentrations of 5-HT led to the desensitisation of the positive inotropic and chronotropic effects of 5-HT [152]. This desensitisation seems to involve G-protein-dependent protein kinases [152]. In cultured mouse colliculi neurons, desensitisation to 5-HT was significant after 5 min (and more effective after prolonged desensitisation times [153]. Mouse colliculi neurons showed, in descending order, mRNA expression 5-HT_4b_-receptors, 5-HT_4a_-receptors and 5-HT_4e_-receptors; this is similar, but not identical, to the expression pattern in the human atrium [153]. Using HEK293 cells as a model, they presented evidence that G-protein-dependent protein kinase (GRK 2) and GRK 5, but not GRK 4 or GRK 6, were required to desensitise 5-HT_4a_-receptors to 5-HT [153]. Similarly to 5-HT_4a_-receptors, 5-HT_4b_-, 5-HT4_e_- and 5-HT_4f_-receptors were also desensitised in transfected COS-7 cells (cells being CV-1 (simian) in origin, and carrying the SV40 genetic material) when GRK2 was coexpressed [153].

At this stage, it is helpful to mention that, in the human heart or the mouse heart, GRK 2 is also abundant, and GRK 5 is at least present (review: [154,155]). Hence, this mechanism might be operative in the human heart. Mutated GRK 2 did not act as a kinase but was still able to desensitise 5-HT_4_-receptors in transfected cells (COS-7) to 5-HT, suggesting that binding to the receptor, but not phosphorylation of the receptor, is the mechanism involved here [153]. The exposure of receptor-transfected cells (HEK293) with 5-HT resulted in cell culture in the binding of the receptors (5-HT_4a,b,e_-receptors were tested) to β_2_-arrestin and internalisation of the dimer of the appropriate 5-HT_4_-receptor and arrestin within minutes, implying uncoupling of these receptors, and thus, their desensitisation [153]. In human and mouse hearts, β_2_-arrestin is expressed [156]. The internalisation seemed to first encompass localisation of the 5-HT_4_-receptors endocytotic vesicles and then the perinuclear membrane [153]. This endocytosis depends upon the intact kinase activity of GRK 2 [153]. Endocytosis of 5-HT_4_-receptors can also be mediated by proteins other than β_2_-arrestins [153].

## 10. Positive Chronotropic Effects of 5-HT (Figure 2, Table 1) and 5-HT_4_-Receptors

5-HT increased the heartbeat rate in isolated atrial preparations of cats, rats, pigs, and guinea pigs [117,122,125,132], as well as in patients [108]. In living instrumented pigs and isolated porcine cardiac preparations, 5-HT elevated the heart rate through 5-HT_4_-receptors [28,112,118]. This increase in the heart rate presumably starts at the 5-HT_4_-receptors followed by stimulatory guanosine-triphosphate-binding proteins (G_s_), adenylyl cyclases (AC), and cAMP, and then depolarises cells via the activation of hyperpolarisation-activated cyclic nucleotide-gated (HCN) channels in the sinus node [112]. More specifically, 5-HT augmented a molecular current called I_f_ in human atrial cells. This current is deemed activated by 5-HT_4_-receptors because the current is attenuated by receptor blockers [26,92,157].

I_f_, the so-called “funny” current, is activated on hyperpolarisation and shows permeability for Na^+^ and K^+^ (Figure 2). It is typically expressed in spontaneously active electrical cardiomyocytes such as sinoatrial cells, AV-nodal cells, and Purkinje fibres. Its open probability is enhanced by cAMP [158]. In more detail, the current–voltage curve of the I_f_, also known as the pacemaker current, is shifted to more negative potentials by the activation of 5-HT_4_-receptors, while its maximum current amplitude remains unaltered [92,157]. This activation was not seen if I_f_ was previously maximally activated by cAMP. It may be assumed that 5-HT can exert a proarrhythmic effect via the activation of I_f_. However, the effect of 5-HT_4_-receptor stimulation is comparable in human atrial cells isolated from patients with sinus rhythm or chronic atrial fibrillation [159].

5-HT was reported to induce cardiac arrhythmias in vivo (tachycardia and P-wave inversions in only two patients [108]. Notably, 5-HT led to arrhythmias, even in isolated human atrial cardiomyocytes [27]. The incidence of arrhythmias was higher in isolated atria from humans pre-treated several weeks before surgery with β-adrenoceptor antagonists [27,91]. The arrhythmias presumably involve late afterdepolarisations [99,101]. These arrhythmias might also result from the activation of L-type Ca^2+^ channels and potassium channels [112]. 5-HT could be essential to maintain pre-existing arrhythmia; during pre-existing atrial fibrillation, more 5-HT should leave thrombocytes [160]. This release is expected to increase concentrations of 5-HT in neighbouring cells, and this 5-HT stimulates 5-HT_4_-receptors to maintain an already existing fibrillation [28]. In children with stimulating autoantibodies for 5-HT_4_-receptors, this may cause AV blocks (e.g., in neonates) [161]. In 5-HT_4_-TG, arrhythmias have been reported under basal conditions or after 5-HT stimulation [36,40,162]. Others further noted an increase in the beating rate due to serotonin in neonatal mouse cardiomyocytes in culture [163].

Another electrophysiological effect of 5-HT has been found on gap junction intercellular coupling. Gap junctions are dodecameric channels connecting cardiomyocytes via a low-resistance electrical pathway. They enable the action potential to spread from one cell to another. Typically, they are found at the cellular poles of cardiomyocytes, thus contributing to the anisotropy of cardiac tissue [164,165]. Interestingly, it was found that gap junction intercellular communication is antagonistically regulated by 5-HT_2_ and 5-HT_4_-receptors [166]. Thus, gap junction currents are enhanced by 5-HT via the stimulation of 5-HT_2A_-receptors and 5-HT_2B_–receptors (in the presence of 5-HT_4_-receptors-inhibitors), but markedly decreased with the stimulation of 5-HT_4_-receptors (in the presence of inhibitors of 5-HT_2_-serotonin-receptors) in neonatal rat atrial cells [166]. Changes in gap junction intercellular coupling alter the biophysical electrical properties of the tissue and contribute to arrhythmogenicity [165].

## 11. The 5-HT_4_-Receptor, in General (Figure 2)

In principle, 5-HT can stimulate several different serotonin receptors. We currently distinguish seven major subtypes, now termed 5-HT_1-7_- serotonin receptors [21,167,168,169]. The 5-HT_3_-receptor sticks out because it acts as a ligand-gated ion channel. Therefore, it does not need to be couple with other proteins to exert its function [169]. All six other 5-HT-receptors belong to the family of heptahelical receptors, and they can all couple to stimulatory (G_s_) or inhibitory (G_i_) guanosine triphosphate-(GTP)-binding proteins [169]. The 5-HT_1_- and 5-HT_7_-receptors diminish the activity of AC via G_i/q_, whereas 5-HT_4_-, 5-HT_5_-, and 5-HT_6_-receptors can augment the activity of AC via G_s_ [169]. The various isoforms of the 5-HT_2_-receptors, via G_q_/G_11_, do not act upon AC, but they can stimulate PLC and elevate IP_3_ levels as well as form diacylglycerol [169]. This diacylglycerol can raise the activity of protein kinase C (PKC). Moreover, at least two subtypes of the 5-HT_2_-receptor, the 5-HT_2A_- and 5-HT_2C_-receptors, can stimulate the activity of phospholipase A_2_ [169].

## 12. Expression of 5-HT_4_-Receptors in Animal Hearts

### 12.1. Mice

Early on, the 5-HT_2b_-receptor was identified by RT-PCR in the mouse myocardium [170]. In later, more complete studies, in the mRNA from a whole adult mouse heart (comprising several cell types), using RT-PCR, one identified 5-HT_1A_-, 5-HT_1B_-, 5-HT_1D_-, 5-HT_2A_-, 5-HT_2B_-, 5-HT_2C_-, 5-HT_3_-, and 5-HT_4_-receptors [33]. In the same study, the 5-HT_6_-receptor was not detected in adult mouse hearts [33]. Others later did not find the 5-HT_4_-receptor in adult mouse hearts, and only reported the expression of 5-HT_2A_- and 5-HT_2B_-receptors [171]. However, the expression of 5-HT_3_-receptors was confirmed in the ventricles of wild-type mice [172]. Five splice variants of the 5-HT_4_-receptor were found in the mouse brain, but only one variant was found in the mouse heart [173]. In contrast, four isoforms of mouse 5-HT_4_-receptors have been cloned [174], and two [174], and later four, splice variants have been described (on RNA level) in mouse atria [175]. The mouse gene for the 5-HT_4_-receptor is located on chromosome 18, comprises at least six exons and five introns with a length of 145 kb, and leads to a protein sequence of 388 amino acids, which is consistent with an apparent molecular weight of 40 kDa [173]. This weight could increase due to post-translational modifications such as phosphorylation, glycosylation, or palmitoylation [169,173].

In adult mouse hearts, there is no contractile response to 5-HT, which would be expected if any 5-HT-receptor, notably the 5-HT_4_-receptors, were functionally present [40]. A caveat is in order. In mouse hearts, the mRNA in the studies referenced here was not prepared from cardiomyocytes, and the atrium and ventricle were not separated. Hence, it would be helpful to investigate mice hearts in more detail, even with (well-characterised) antibodies, and compare them with knockout hearts as negative controls for the antibodies. As in rats (Section 12.2), the expression of mRNA for the 5-HT_4_-receptors was highest in the foetal heart and declined with ageing. The expression of the 5-HT_4_-receptor was minimal at birth in the neonatal mouse heart. Using immunohistology with antibodies raised against the 5-HT_4a_-receptor and the 5-HT_4b_-receptor led to similar findings: on the protein level, high levels of these receptors in the mouse atrium and ventricle were microscopically visible after staining with appropriate antibodies. However, they disappeared after the birth of the mice [176].

### 12.2. Rats

Interestingly, the 5-HT_4_-receptor cDNA was first described in rats by Gerald et al. (1995) [177]. They noted two splice products, which they called S and L. In the adult rat ventricle, using RT-PCR, they failed to detect any 5-HT_4_-receptor, while in the atrium of the rat, they found only one transcript of the 5-HT_4_-receptor that they later called the S 5-HT_4_-receptor [169]. However, the role of 5-HT_4_-receptors in rat hearts is quite complicated. 5-HT_4_-receptors as well as 5-HT_2A_-receptors are expressed as mRNA in rat hearts [80]. Both receptors were expressed in the atrium as well as in the ventricle of the rat heart [80]. Only in the isolated rat atrium, but not in rat ventricular preparations, did 5-HT exert a positive inotropic effect; this effect in adult rat atrial preparations was mediated via the 5-HT_2A_-receptor, but not via the 5-HT_4_-receptor; this was quite unexpected for the investigators at that time [80]. The expression of 5-HT_4_-receptors on protein (at 72 kDa) and mRNA [166] in rat neonatal cardiomyocytes has been reported. More specifically, the expression at mRNA levels was about 20 times higher for 5-HT_4b_-receptors than 5-HT_4a_-receptors [166]. These data are valuable because mRNA coding for 5-HT_4_-receptors was observed in earlier work. However, the RNA was extracted from whole tissue (atrium or ventricle) and will undoubtedly have contained, to some extent, mRNA from non-cardiomyocytes [80].

Interestingly, 5-HT alone or in the presence of isoprenaline reduced cAMP levels in neonatal rat auricular cardiomyocytes [166]. These effects of 5-HT were attenuated by a 5-HT_4_-receptor antagonist, and therefore, 5-HT_4_-receptor-mediated [166]. Fitting to the reduction in cAMP, 5-HT in neonatal rat auricular cardiomyocytes reduced the current through L-type Ca^2+^ channels [166]. The authors argued that the expression of the inhibitory G-protein is known to decline from neonatal to adult rat hearts [166]. Thus, the inhibitory action might mirror these changes. In other words, only in auricular neonatal cardiomyocytes is the expression of inhibitory G-proteins high enough to couple between AC and 5-HT_4_-receptors, leading to a cAMP reduction that vanishes in ageing [166]. Whether the same holds true in the human heart remains to be seen. The expression of the cardiac rat 5-HT_4_-receptor is age-dependent. There is a high expression of the 5-HT_4_-receptor in the foetal rat heart that declines upon birth and into adulthood [130,178]. Therefore, analogous to atrial natriuretic peptides (ANF), one has suggested that the cardiac expression of the 5-HT_4_-receptor follows a foetal gene programme [169]. Whether the expression of the 5-HT_4_-receptor in the human foetal heart is higher than in the adult human heart is unknown, but such a decline has also been described for the human cardiac D_1_-dopmine receptor.

As mentioned above, the expression of the rat heart 5-HT_4_-receptor increases after experimental cardiac hypertrophy, experimental hypertension and heart failure after inducing myocardial infarction [127,128,179,180]. Later, it was confirmed that 5-HT_4_-receptors are expressed at the mRNA level and on the protein level in rat neonatal cardiomyocytes [163]. They extended previous studies [179] by describing the presence of 5-HT_4_-receptors in mitochondria from rat neonatal cardiomyocytes [163]. The role of 5-HT_4_-receptors in mitochondria opens an interesting intellectual challenge. First, as mitochondria are located within cardiomyocytes, the agonist is probably cytosolic serotonin in the cardiomyocytes (known to exist; see above). One can postulate that under conditions of energy need, serotonin concentrations in cardiac cytosol increase, and this signal is conferred to the mitochondria utilising 5-HT_4_-receptors. They would now increase, via G_s_ and AC, the levels of cAMP and activated cAMP-dependent protein kinase and phosphorylate, thereby activating enzymes for adenosine triphosphate (ATP) synthesis in the cardiac mitochondria (Figure 2). However, this entire pathway needs to be proven experimentally. Moreover, these data clarify that the positive inotropic effects observed in rat neonatal ventricular cardiomyocytes were induced by giving 5-HT to these cardiomyocytes and not indirectly, as in indirect sympathomimetic agents such as amphetamine that simply release noradrenaline from cardiac stores but are inactive in the presence of cocaine [178]. They also confirmed and extended previous work, which concluded, in contrast to neonatal ventricular cardiomyocytes, that the 5-HT_4_-receptors in adult ventricular cardiomyocytes do not lead to inotropic responses in the ventricle.

### 12.3. Pigs and Monkeys

As in smaller mammalian species, the 5-HT_4_-receptors display at least 11 splice variants in pigs [181]. Their role is still uncertain. The splicing borders of the 5-HT_4_-receptor isoforms in pigs are different from those in humans. Hence, one might question whether pigs are an effective model for testing the function of new agonists or new antagonists at the 5-HT_4_-receptor that are intended to be used in humans [181]. However, this is regularly performed in the pharmaceutical industry because the size and physiology of the pig heart show many similarities to humans. Physiological methods for pigs are very well established in the industry, and the industry lacks other models, and perhaps access to clinical samples. The porcine 5-HT_4_-receptor gene is located in porcine chromosome 2 [181]. The amino acid sequence leads to products composed of 369 to 412 amino acids [181]. The function seems to vary somewhat based on the splice variant studied. For instance, prucalopride was more effective in raising cAMP (in transfected cells, thus an artificial environment) than 5-HT, in what they termed porcine 5-HT_4a_-receptor [181]. 5-HT_4_-receptors were first found in the left and right atria of the pig [182], and later in the porcine ventricle [181]. They convey a positive inotropic and chronotropic effect to serotonin when serotonin alone was applied in the organ bath (atrium, [90,118,141,146]. These contractile effects of serotonin could be amplified by the additional presence of phosphodiesterase inhibitors (ventricle: [112,146,179,182]) (see Table 1). The mRNA for the 5-HT_4_-receptor was detected at about the same level in the monkey atrium and ventricle [140]. Any splice variants of the 5-HT_4_-receptor in monkeys were not reported and might not exist [140].

## 13. Expression of Serotonin Receptors in the Human Heart

On the RNA level, 5-HT_4a_- and 5-HT_4b_-receptors in the first studies were described in the human atrium [182,183] and also in the human cardiac ventricle [179]. Both mRNAs for the 5-HT_4a_-receptor and the 5-HT_4b_-receptor were expressed in the left atrium, right atrium, left ventricle, and right ventricle of the human heart in post-mortem samples, suggesting the remarkable stability of this mRNA [182]. At least 11 splice variants of the human 5-HT_4_-receptor are known. In the human atrium (a, b, c, g, i, and n), more splice variants are expressed than in the human ventricle (a, b, g, and i) for unknown reasons, and are functionally not yet fully understood [184]. The human gene coding for the 5-HT_4_-receptor is located on chromosome 5 [185] and is reported to contain a minimum of 14 exons. Initially, splicing in the cytosolic C-terminal sequence of the human 5-HT_4_-receptor was reported later in the brain. In addition, splicing in the N-terminal sequence of the human 5-HT_4_-receptor was described [169]. The predicted sequences for the human 5-HT_4_-receptor range from 359 to 428 amino acids [169]. This suggests a mean molecular size of about 40 kDa on Western blotting for the monomer.

However, outside the cardiomyocyte, the 5-HT_2A_-receptors have been detected in human arterial smooth muscle cells; there, they can induce vasoconstriction [123,186]. The 5-HT_4_-receptor (but not, for instance, a 5-HT_2A_-receptor) is responsible for the positive inotropic effect and positive chronotropic effect in the human heart [59,89,96]. Strong antibodies to 5-HT_4_-receptors or splice variants are not available. Hence, the protein levels of these receptors are difficult to measure. However, some radioactive ligand binding studies have made it feasible to measure the protein expression levels in the heart and found minute densities of 5-HT_4_-receptors in the heart [97,187]. 5-HT_1_-receptors were also found in endothelial cells and smooth muscle cells from human coronary arteries, which may lead to vasoconstrictory effects of 5-HT [186] and can reduce AC activity [188]. 5-HT_2B_-receptors were found mainly in cardiac valves. Their stimulation by 5-HT, fenfluramine (indirectly by inhibiting SERT or by releasing 5-HT from platelets), ergotamine-derivatives, methysergide, and recreational drugs (“ecstasy”) is thought to have caused deadly valve ruptures [189,190,191]. These drugs can cause valve dysfunction in the right and left atria (review: [145]). Another layer of complexity in human disease that we would predict will come from a better understanding of splice variants, and possibly, mutants of the 5-HT_4_-receptor. There seems to be a consensus that all splice variants of the 5-HT_4_-receptor stimulate cAMP levels in transfected non-cardiac cells (e.g., hamster embryonic kidney cells: HEK cells). Looking carefully at the data [179], these seem to be small, but potentially clinically relevant changes in the efficacy or potency of 5-HT to stimulate an increase in cAMP under these conditions. There is evidence from point mutations performed in vitro that some mutated 5-HT_4_-receptors couple only to the generation of IP_3_ or cAMP [192]. For the human 5-HT_4a_-receptor, some mutational data indicate where the binding site of 5-HT might be localised [193].

## 14. Signal Transduction of 5-HT_4_-Receptors, in General

When we now focus on signal transduction of the 5-HT_4_-receptor in general, and then dwell on its action in the hearts of experimental animals and then in humans, the following outline emerges (Figure 2): the 5-HT_4_-receptor (in all its splice variants tested thus far in transfected cells) can activate the AC via Gs, and hence increase (in most cases) cellular cAMP content (review: [169]. This led to the functional description and the claim of the existence before cloning of the 5-HT_4_-receptor as a cAMP-increasing receptor in the brain [194,195] and later in the human heart [59,89]. Overexpressed 5-HT_4_-receptors in transfected cells and transgenic mice are constitutionally active [40,174]. The shorter the slice variant of the 5-HT_4_-receptor, the higher its activity to increase cAMP levels in transfected cells [174].

When stimulation of the 5-HT_4_-receptor generates cAMP, this cAMP can now activate the cAMP-dependent protein kinase (PKA). PKA can phosphorylate target proteins (ion channels, phospholamban (PLB), or the inhibitory subunit of troponin (TnI) in the present context) and activate them (Figure 1 and Figure 2). PKA can also bind to PKA-anchoring proteins (AKAPs), at least in neuronal cells, upon activation of the 5-HT_4_-receptor (rat neurons: [196]. This AKAP-binding is likely to occur via the 5-HT_4_-receptor in the heart because this pathway is known to exist as a consequence of the activation of cardiac β-adrenoceptors [197]; however, this needs to be experimentally proven.

The generated cAMP can also directly bind to the cAMP-dependent exchange protein activated by cAMP (EPAC). Any β-adrenergic stimulation is known to activate EPAC in the heart [198]. EPAC binding upon the activation of 5-HT_4_-receptors was reported in neuronal cells or transfected cells [199], and needed to be shown in cardiomyocytes. The generated cAMP can be degraded by cardiac phosphodiesterases, but can also activate or inhibit cardiac phosphodiesterases.

The 5-HT_4b_-receptor, but not 5-HT_4a,d_-receptors, can inhibit the activity of AC via Gi (Figure 2), seldom reducing the cellular cAMP content in cardiac cells (rat neonatal cardiac cells: [166], failing adult rat ventricle: [200]). Whether such events take place in human hearts in neonates or in adult human heart failure might be relevant for further research and might be of clinical relevance.

The 5-HT_4_-receptor can activate the enzyme PLC via G11,q, and thereby generate diacylglycerol (DG) and inositoltrisphosphate (IP_3_). However, this pathway has previously only been noted outside the heart; for instance, in human 5-HT_4_-receptor-desoxyribonucleid acid (DNA)-transfected COS cells, a very unphysiological system that is useful only as a proof of principle [192,201]. Using these transfected cells, it was possible to derive a point mutation of the 5-HT_4_-receptor (D100A), in which 5-HT, in contrast to other agonists, failed to raise IP_3_ levels. Hence, using appropriate drugs, it might be possible to stimulate only the IP_3_, but not the cAMP pathway. Then, using these drugs in contraction experiments in the human atrium, one might be able to dissect how much IP_3_ or cAMP contributes to the positive inotropic effect of force generation. In addition, further functions of 5-HT_4_-receptors in addition to force generation in the heart might be studied with such biased agonists [201]. At least in the rat heart, only the 5-HT_2A_-receptors activated phospholipase C and raised IP_3_-levels [80].

Activation of extracellular-receptor-coupled kinases (ERK) in transfected cells does not follow the pathway via cAMP or PLC (review: [202]). ERK was not activated via G-proteins or β-arrestin (review: [202]). ERK is activated via the phosphorylation and activation of a tyrosine kinase called Src (review: [202]). Activated Src then activates PLC (review: [202]). This was observed in neuronal cells, HEK cells, and enterocytes [153,203]. It is plausible that this pathway is also used in the human heart or even in human cardiomyocytes; however, this needs to be demonstrated experimentally. For instance, Src is present in the human heart (e.g., [204]). One would also wonder to what extent this pathway increases the force of contraction compared with the increase in cAMP. This issue might be addressed by applying selective inhibitors or genetic approaches in this signal transduction pathway.

Theoretically, the 5-HT_4_-receptor might directly stimulate or inhibit ion channels. Most evidence is contrary; 5-HT_4_-receptors act on ion channels via increased cAMP content. Nevertheless, a direct interaction of 5-HT_4_-receptor and ion channels is a logical research aim in the heart.

Signal transduction of the 5-HT_4_-receptor via phosphatases is an exciting but complicated topic (Figure 2). In murine colliculi neurons, stimulation of 5-HT_4_-receptors led to the inhibition of a potassium ion current [205]; this inhibition was potentiated by 10 nM okadaic acid. The authors’ interpretation indicated an involvement of PP1 or PP2A [205]. This result is relevant but needs confirmation using, for instance, animals with a genetic manipulation of PP1 and PP2A, because at 10 nM okadaic acid, even in vitro, PP1 is not inhibited, only PP2A (review: [206]). Hence, it remains open, especially in the mammalian heart, which phosphatase subtype is involved. Hence, one can postulate a direct coupling of the 5-HT_4_-receptor to phosphatases. Alternatively, or additionally, more classical pathways are likely, but unexplored. Similarly to the stimulation of β-adrenoceptors, stimulation of the 5-HT_4_-receptor is known to activate PKA in the human heart (right atrium: [89]). Stimulation of β-adrenoceptors led to an increased phosphorylated state of phosphatase inhibitor-1 (isolated guinea heart: [207]) or DARPP32 (brain: [208]). A phosphorylated phosphatase inhibitor-1 or DARPP32 would now be activate and could inhibit the activity of PP1 (review: [206]). In summary, amplification of the response to PKA ensues. Increased phosphorylation states of phosphatase inhibitor-1 or DARPP32 by stimulation of 5-HT_4_-receptors in any tissue, especially the human heart, remains to be reported.

## 15. Signal Transduction of 5-HT_4_-Receptors in Animal Hearts

In general, the signal transduction of the 5-HT_4_-receptor was first shown to encompass the stimulation of AC via Gs and thereby increase cAMP formation. The 5-HT_4_-receptors in rat hearts lead only to positive inotropy after experimental myocardial infarction or experimental hypertension [146]. The 5-HT_2A_-receptors, at least in the rat heart, seem to activate phospholipase C and raise IP_3_-levels [80].

Under these conditions, prucalopride failed to affect contractile function in adult rat cardiac cells. The 5-HT_4_-receptor couples to intracellular pathways via P11 (Figure 2). There is evidence of this pathway in the brain and the heart [209]. P11 is a protein that interacts with G-proteins; P11 is also a Ca^2+^ binding protein [210]. Indeed, when P11 expression was augmented by the incubation of adult rat cardiomyocytes for 8 h with 50 ng/mL brain-derived-neurotrophic factor, 1 µM prucalopride exerted a positive inotropic effect, increased free cytosolic Ca^2+^ levels, and an increased incidence of spontaneous calcium ion releases (which indicates a ventricular arrhythmia). These effects were blocked by 10 µM GR113808 and were thus regarded as 5-HT_4_-receptor-mediated [209]. Moreover, as imipramine, a serotonin uptake inhibitor used to treat depression, could increase P11 levels, rats were treated for one week with imipramine (intraperitoneally for 21 days); similarly, the expression of P11 in the heart increased, and 1 µM prucalopride was able to increase contractility and calcium transients and induce arrhythmias in isolated adult rat ventricular cardiomyocytes [209]. The treatment of living rats with brain-derived neurotrophic factor for 14 days led to positive chronotropic effects via the 5-HT_4_-receptor in living animals, as measured by ECG [209]. This suggests that P11 can modulate the function of the 5-HT_4_-receptor in the mammalian heart in vivo in several ways. For instance, p11 might facilitate coupling efficacy of the 5-HT_4_-receptor to increase cAMP (which was not reported), or P11 might increase the expression of the 5-HT_4_-receptor (which was not reported). The 5-HT_4_-receptor may lead to the translocation of P11 to other subcellular compartments within the cardiomyocytes (Figure 2), Interestingly, P11 was expressed in the adult rat heart; the expression of P11 was higher in the rat atrium than in the rat ventricle. Notably, P11 was also found in rat ventricular cardiomyocytes on mRNA and protein levels [209]. At least in human coronary arteries, P11 (S100A10) was detected [211], suggesting that these data on rats might be translatable to human cardiomyocytes; however, this must be thoroughly studied.

As mentioned above, interventions such as experimental hypertension and subsequent heart failure increase the expression and the inotropic function of the 5-HT_4_-receptor in the rat ventricle [212]. The authors rightly suggested that their data might partly explain why imipramine can lead to arrhythmias in patients [209]. On the other hand, one must remember that imipramine, as a serotonin reuptake inhibitor, can potentiate the cardiac effects of serotonin in the human atrium, similar to the described mechanism of cocaine [59]. An interesting experiment might be to repeat this study using mice with cardiac-specific KO of P11 or the knockdown of P11 in rat myocytes with antisense RNA. Then, one could prove or disprove the role of P11 in this context.

There seems to be a consensus that adult rat cardiomyocytes usually do not express functionally active 5-HT_4_-receptors. This observation was used to study 5-HT_4_-receptor isoforms better. To this end, 5-HT_4_-receptor isoforms were expressed using an adenoviral system in cultivated adult cardiomyocytes. It turned out that, although 5-HT could not stimulate the current through the L-type Ca^2+^ channels in non-transfected cells, the adenovirally transfected receptors after the addition of 5-HT were able to enhance this current [142]. Interestingly, at least under these somewhat artificial conditions, signal transduction differences were noted. Through pertussis toxin treatment, a method to inactivate the function of Gi/o-proteins, the effect of 5-HT on 5-HT_4b_-receptors to enhance current through the L-type Ca^2+^ channels was augmented. Such augmentation was lacking in transfected 5-HT_d_-receptors [142]. This might mean that PTX-sensitive G proteins, under some conditions, can break down the action of 5-HT_4_-receptors. One would predict that this should also lead to a diminished positive inotropic effect under such conditions. It would be interesting to perform such experiments with human cardiomyocytes.

Interestingly, and in line with studies in transfected rat cardiomyocytes, in the failing rat ventricle (where the 5-HT_4_-receptor becomes functional, see above), pre-treatment of rats with pertussis toxin increases the positive inotropic effect of 5-HT via 5-HT_4_-receptors in rat papillary muscle [200]. This could mean that the 5-HT_4_-receptor upregulated in heart failure now couples to both G- and PTX-sensitive G-proteins, conceivably Gi. This coupling to Gi diminished the maximum response to 5-HT via Gs on cAMP formation and subsequently to force [200].

More than 150 proteins can physically interact with the 5-HT_4_-receptor in the brain [213]. This kind of interaction should be studied in animal and human cardiomyocytes, which would clarify what direction one should pursue in understanding new signal transduction mechanisms in the human heart. This cascade should ultimately also increase Ca^2+^ transients in cardiomyocytes and the force of contraction.

## 16. Signal Transduction of 5-HT_4_-Receptors in Human Hearts

Regarding signal transduction, 5-HT increased cAMP content. The activity of PKA in the human heart [89] and 5-HT increased the phosphorylation state of phospholamban (PLB) and the inhibitory subunit of troponin (TnI) [70] in the human heart. These effects were attenuated by the 5-HT_4_-receptor antagonists [70]. Hence, these effects were probably 5-HT_4_-receptor-mediated in the human heart [70]. 5-HT raised the current through the L-type Ca^2+^ current in the human atrium [81,85,88,90], but not the human ventricle [81]. In multicellular preparations from the human atrium [59], 5-HT increased the force of contraction, but 5-HT also increased contractility in isolated electrically stimulated human adult atrial cardiomyocytes [91]. 5-HT in 5-HT_4_-TG induced a positive inotropic effect and positive chronotropic in intact mice, in their isolated perfused hearts, in their isolated left atria (electrically driven), or in their isolated spontaneously beating right atrium [40]. These effects in various preparations from 5-HT_4_-TG led to cAMP increases, an increased phosphorylation state of PLB (on amino acid serine 16 and threonine 17), and an augmentation in the current through L-type Ca^2+^ channels. 5-HT elevated the free Ca^2+^ content in the cytosol in ventricular preparations or whole hearts from 5-HT_4_-TG [40]. 5-HT also elevated the phosphorylation of PLB in atrial preparations from 5-HT_4_-TG [214]. In addition, the in vivo activity of agonists could be studied on contractility [109]. 5-HT could desensitise the 5-HT_4_-receptor in the 5-HT_4_-TG [109].

Interestingly, there is an interaction between 5-HT_4_-receptors and other G_s_-coupled receptors. In transgenic mice that overexpressed both human H_2_- and 5-HT_4_-receptors, but more importantly, also in human atrial preparations, when a concentration–response curve for the force of contraction was first constructed, subsequently applied histamine reduced the force of contraction [102]. This was interpreted in the following way: 5-HT_4_-receptors only couple to G_s_, whereas H_2_-histamine-receptors can couple not only to G_s_, but also to G_i_ proteins [102]. This coupling to G_i_ might occur at low concentrations of histamine, whereas at higher histamine concentrations, the stimulation of G_s_ prevails [102]. This might be clinically relevant, because histamine is also formed in the human heart. This histamine, via the H_2_-histamine receptor, might act as a brake in the 5-HT_4_-mediated stimulation of the human heart. However, this needs to be tested in patients in clinical trials [102]. Using specific phosphodiesterase inhibitors, one could show that, in 5-HT_4_-TG, the inotropic effects of 5-HT are mediated by PDE 2 and PDE 4 [110].

Using a fluorescent-labelled cAMP binding protein as a read out, it could be directly shown that 5-HT via 5-HT_4_-receptors leads to a local cytosolically located increase in cAMP levels in human atrial cardiomyocytes [215]. This result is an important step forward in defining the exact signal transduction mechanism in the human heart on a subcellular level. It is awaited with interest whether, with other labelled proteins, it will become possible to measure 5-HT_4_-induced cAMP, such as near the sarcoplasmic reticulum, the mitochondria, or even nuclear membranes in human cardiomyocytes. Using similar approaches, it will also be possible to study cAMP-independent pathways of 5-HT_4_-receptors in the human heart on a subcellular level, which is a prerequisite to devising novel cardiovascular drugs. Notably, these authors found that cAMP elevation due to 5-HT could be augmented by the PDE inhibitors rolipram and cilostamide in an additive fashion [215]. Moreover, they could confirm and extend their previous studies that in chronic atrial fibrillation in patients, the inotropic effect to 5-HT was diminished, and this attenuated a positive inotropic response to 5-HT via 5-HT_4_-receptors, accompanied by, and likely explained by a diminished increase in cellular cAMP levels [215].

Serotonin increased the Ca^2+^ transients in human atrial cardiomyocytes, the currents through the L-type Ca^2+^ channels, the phosphorylation state of phospholamban, the phosphorylation state of TnI, and the phosphorylation state of the myosin-binding C-protein in human atrial preparations (from patients in sinus rhythm, [104]). Notably, the maximum increases in the phosphorylation state of phospholamban and the phosphorylation state of the troponin inhibitor after 5-HT_4_-receptor stimulation were much lower than those of β-adrenergic stimulation, consistent with fewer increases in cAMP and PKA activity under these conditions [104].

In human atrial preparations, serotonin (10 µM), the maximal effective inotropic concentration, increased cAMP in the left [87] and right atrial preparations [89]. The maximum inotropic effective isoprenaline concentration elevated cAMP in the right and left human atrial preparations to a much higher extent [87]. This was consistent and, therefore, might explain the observation that isoprenaline was much more effective in raising the force of contraction than serotonin in the human left atrium to 24.5% [87]. In contrast, the effect of 5-HT on right atrial preparations amounted to about 19% of the maximum effect of isoprenaline [91]. On the one hand, this means that the effectiveness of serotonin correlates nicely with its ability to raise cAMP levels. On the other hand, this observation reveals a gap in our understanding of the signal transduction of the 5-HT_4_-receptor. If both receptors coupled to the same signal transduction pathway, namely, stimulatory GTP-binding protein (G_s_), stimulating the activity of AC, their potency and efficacy on cAMP levels might be identical.

This is not the case. Hence, the signal transduction of isoprenaline and serotonin must diverge, possibly accounting for these differences. However, how exactly and to what extent the divergent signal transduction mechanisms of 5-HT_4_-receptors might impair cAMP generation compared with the full agonist isoprenaline appears to be an interesting remaining problem in the field. Others have noted that 5-HT_4a_-receptors are predominant in human atrial homogenates [140]. There seems to be a difference between broken cell preparations and intact cells. In broken cell preparations, 5-HT was less potent in raising cAMP levels [182] than in intact transfected cells [216], suggesting methodological differences. These differences were used to explain why cisapride and prucalopride were more effective in raising cAMP levels in intact cells [216] than in broken cell preparations [182].

## 17. 5-HT_4_-Receptors in Cardiac Disease

### 17.1. Heart Failure

There is consistency in the literature that higher plasma serotonin levels accompany heart failure. For instance, in a recent study independent of age or medication, stable heart failure led to higher plasma serotonin levels than normal control patients [217]. Moreover, serotonin levels in plasma increased as the New York Heart Association (NYHA) class of heart failure increased [217]. For instance, the basal value of serotonin in non-heart failure patients was 0.76 ng/mL (n = 17), increasing to 1.91 ng/mL (n = 156) in patients with heart failure. In NYHA IV, a value of 5.25 ng/mL was reported [217]. The authors suggested that this increase in the plasma levels of serotonin might present a compensatory mechanism.

An antagonist at the 5-HT_2A_-receptors diminished cardiac hypertrophy after banding in mice [218]. This may imply the role of this receptor in cardiac hypertrophy [218]. This hypertrophy was accompanied by an increase in the expression of the 5-HT_2A_-receptors [218]. Isoproterenol-induced hypertrophy in mice was diminished in those treated with a 5-HT_2B_- blocker or in 5-HT_2B_-receptor knockout mice. This was explained by reduced peroxide generation in cardiac mitochondria [219,220]. Such isoproterenol-mediated cardiac hypertrophy requires 5-HT_2B_-receptors on cardiac fibroblasts [221]. Consistent with this concept, in patients with cardiac hypertrophy, the expression of 5-HT_2B_-receptors was elevated [221]. These 5-HT_2B_-receptors were identified using immunohistology in human cardiomyocytes and human non-cardiomyocytes in the heart (mainly fibroblasts). Whether elevated expression of these receptors in human heart failure occurs in cardiomyocytes or in non-cardiomyocytes of the heart remains an open question [221].

In heart failure, 5-HT is elevated in the plasma of patients with decompensated systolic heart failure [217] or diastolic heart failure [222]. It has been speculated that such a 5-HT elevation might be a compensatory mechanism in heart failure in the effort to increase heart rate and cardiac force [217]. In atrial muscle strips from heart failure patients, the positive inotropic effect of 5-HT was attenuated. Likewise, 5-HT raised AC activity less [95] or augmented L-type Ca^2+^ currents less in samples from heart failure patients [85]. Some of these effects were absent after prolonged treatment with β-adrenergic antagonists in patients before the operation [223]. When experimental infarcts were induced in a rat model of heart failure, the mRNA of 5-HT_4_-receptors increased, and a robust positive inotropic effect of 5-HT via 5-HT_4_-receptors (which was missing in healthy rats) became apparent [127]. However, there might be species and regional differences.

The positive inotropic effect of 5-HT was higher in patients with terminal heart failure [84]. Piboserod, a 5-HT_4_-receptor antagonist, reduced cardiac hypertrophy in heart failure patients [224]. Possibly, 5-HT_4_-receptors might be detrimental to chronic human heart failure. In acute heart failure, the situation might be different. In lipopolysaccharide (LPS)-induced sepsis (a model of acute heart failure), the overexpression of 5-HT_4_-receptors was supposedly beneficial by interference with the Toll-like receptor 4 pathway [225]. In 5-HT_4_-TG, LPS reduced the expression of 5-HT_4_-RNA, suggesting a connection between sepsis and 5-HT_4_-receptor function in the heart [225]. When hypertrophy was induced genetically, 5-HT_4_-TG was cross-bred with mice with overexpression of the catalytic subunit of protein phosphatase 2A (PP2A-TG). This led to greater improvements in diastolic function in double-transgenic mice than in PP2A-TG. Furthermore, it might be interpreted as a protective role of 5-HT_4_-receptors in some forms of hypertrophy [225].

### 17.2. Hypertension

In hypertensive patients, an elevated plasma level of 5-HT has been reported. This is to be expected, as the search for plasma compounds elevated in hypertension was the method to find serotonin in the first place [2]. Elevated serotonin in the plasma might have led to the covalent modification of protein rab4. Therefore, the function of SERT in platelets is reduced. Thus, a circulus vitiosus might start because the uptake of 5-HT out of the plasma into the platelets is diminished, and more 5-HT might cumulate in the plasma [226]. There is evidence that 5-HT can inhibit the function of small G-proteins by inducing the serotonylation of these proteins. Here, the function of smooth muscle cells in pulmonary arteries can be impaired, increasing the vasoconstrictory action of 5-HT, especially in lung arteries, which may culminate in pulmonary hypertension, a potentially deadly disease [74,227]. The serotonylation of several proteins occurs in rat aorta [228], such as histones or sarcoplasmic reticulum Ca^2+^-adenosine triphosphate (ATP)-ase (SERCA2a) in cardiomyocytes (review: [73]).

Moreover, there is a wealth of information that serotonin may contribute to adult pulmonary hypertension [229] and neonates [230,231]. One must keep in mind that the effects of serotonin on vascular resistance, and thus, serotonin’s role in the development of hypertension are, at least in part, sex-hormone-related [232]. The role of serotonin in hypertension, namely, pulmonary hypertension, seems to rely solely on vasoconstriction. However, serotonin might harm the vessels by facilitating thrombosis, intravascular coagulation, and local and general inflammation [233]. There also seems to be a genetic link between SERT mutations and pulmonary hypertension [234].

Hypertension can also result from “serotonin syndrome”, a long-known muscular hyperexcitability disease probably due to the drug-induced stimulation of 5-HT_2_-receptor subtypes following medication with serotonin reuptake inhibitors [234]. More specifically, animal studies imply an increase in the expression and function of 5-HT_4_-receptors in L-NAME-induced hypertension. The underlying mechanism seems to involve 5-HT_4_-action in peripheral nerves to release noradrenaline [235]. Moreover, as predictable from anatomy, pulmonary hypertension will lead slowly, although quickly in some animal studies, to right ventricular failure, because now the afterload of the right ventricle suddenly imposes a severe burden [236]. More recently, it has been suggested that the gut microbiome is a source of 5-HT. 5-HT from gut bacteria may be resorbed into the circulation, and thus may cause, at least in part, systemic or pulmonary hypertension [20,237].

### 17.3. Arrhythmias

There is some evidence that, in humans, plasma autobodies can be present that stimulate human 5-HT_4_-receptors. It has been suggested that these autoantibodies might lead to atrial fibrillation [176,178]. Interestingly, antibodies in rabbits and mice have been raised that are antagonistic functionally at human 5-HT_4_-receptors, at least in cell culture [238,239]. This may mean that generating antagonistic antibodies as drugs to suppress atrial fibrillation would be possible. Autoantibodies against 5-HT_4_-receptors have been claimed to play a role in congenital heart block [240].

Cisapride led to arrhythmias in 5-HT_4_-TG [162], similar to arrhythmias noted in patients taking cisapride (215 Olsson et al. 1992). In contrast, 5-HT was less potent than prucalopride, but equally effective [162]. Cisapride, a partial agonist at 5-HT_4_-receptors, can induce tachyarrhythmias in patients [241,242,243]. These tachyarrhythmias might release a preformed thrombus from the right atrium wall, leading to brain insults [244]. It has been suggested that 5-HT, via the activation of cardiac 5-HT_4_-receptors and subsequent formation of cAMP and increased levels of free cytosolic Ca^2+^, might lead to arrhythmias in susceptible patients [28]. The infusion of 5-HT in humans could induce cardiac arrhythmias [107]. This 5-HT-induced induction of arrhythmias can be recapitulated with 5-HT in 5-HT_4_-TG [162]. However, one cannot induce arrhythmias to a greater extent with cisapride in 5-HT_4_-TG compared with WT [162]. This finding argues against the hypothesis that cisapride induces arrhythmias via 5-HT_4_-receptors, but seems to indicate that other mechanisms, most likely inhibiting cardiac potassium ion currents, are the culprit [162].

Likewise, although prucalopride or metoclopramide induced tachycardia in 5-HT_4_-TG, they failed to induce more arrhythmias in 5-HT_4_-TG compared with WT, conceivably due to the protective ancillary mechanisms of prucalopride and metoclopramide [162,245]. The 5-HT_4_-induced arrhythmias seem to reside, at least to a certain extent, in the cardiomyocytes themselves and do not, for instance, come from noradrenaline released from intracardiac stores in ganglia or 5-HT released from cardiac ganglia. This could be concluded from experiments that induced arrhythmias in isolated cardiomyocytes through the application of 5-HT [40,91]. The inhibition of the gap junction intercellular current by 5-HT_4_-receptor stimulation may contribute [166], as well as the shift of the I_f_ IV curve [92]. The incidence of 5-HT-induced arrhythmias was increased by the pre-treatment of patients before surgery with β-adrenoceptor agonists when measuring the force in isolated muscle strips or cell edge detection in isolated cardiomyocytes [24,91,223]. In isolated atrial cardiomyocytes from patients with atrial fibrillation, the 5-HT-induced current through the L-type Ca^2+^ channel was elevated, the duration of the action potential was prolonged, and 5-HT was more often induced afterdepolarisation [100]. In addition, 5-HT can augment the I_f_-current in sinus node cells [26,92].

Interestingly, there are autoantibodies in patients, notably children, against 5-HT_4_-receptors, which are thought to lead to rhythm disturbances [161,246]. The porcine heart contains 5-HT_4_-receptors [181]; one has failed to induce 5-HT any cardiac arrhythmias in pigs. This has been suggested to be due to the lower density of 5-HT_4_-receptors in pigs compared with humans [93,96]. This seems to emphasise the potential role of 5-HT_4_-TG in assessing the mechanism of 5-HT-induced arrhythmias in patients. The expression of the 5-HT_4_-receptor mRNA levels was reduced in patients with atrial arrhythmias and might be a protective mechanism [247]. The incidence of arrhythmias at 37 °C in the organ bath was higher in right atrial preparations from the 5-HT_4_-TG compared with WT under basal conditions [162,214]. This is usually explained by the constitutive stimulation of the overexpressed 5-HT_4_-receptor to the activity of AC and the subsequent production of cAMP and elevation of cardiac Ca^2+^ levels. This increase in Ca^2+^ could lead to more arrhythmias in 5-HT_4_-TG than in WT. In addition, endogenous 5-HT might activate AC, contributing to an increased incidence of arrhythmias even under nominal basal conditions [29]. This might be of clinical relevance, because 5-HT levels are sufficiently high in the human atrium to stimulate 5-HT_4_-receptors in the human heart [29].

On the other hand, if patients are treated with drugs that are agonists at 5-HT_4_-receptors, the incidence of arrhythmias is also expected to increase. This is the prediction of other studies. Exogenous 5-HT increased the incidence of arrhythmias of right atrial 5-HT_4_-TG [162]. Interestingly, hypothermia can also affect 5-HT_4_-mediated arrhythmias. In right atrial preparations from the 5-HT_4_-TG, the incidence of arrhythmias increased significantly more than in WT used as controls [111]. This was accompanied by, and might be due to, a higher overexpression of heat shock proteins of the apparent molecular weight of 70 kilo Daltons (HSP70) in right atrial preparations from the 5-HT_4_-TG than WT [111].

The constitutive knockout of all 5-HT_3_-receptors in mice was accompanied by spontaneous ventricular tachycardia and sudden death in pregnant mice. Therefore, 5-HT_3_-receptor antagonists might be dangerous in pregnant patients [172]. Furthermore, ondansetron might elicit arrhythmias in patients [248]. Moreover, arrhythmias in the form of prolonged P-waves and highly elevated T-waves were observed in mice with the deletion of 5-HT_2B_-receptors [249].

Another interesting indirect mechanism in the experimental literature is why 5-HT can cause arrhythmias in humans. Specifically, the long-term treatment of neonatal rat cardiomyocytes in culture with 5-HT increased the expressional level of connexins in these cells [166]. Acutely, 5-HT can increase intercellular electrical coupling via 5-HT_4_-receptors in rat neonatal cardiomyocytes, possibly contributing to arrhythmias [166]. However, in atrial neonatal rat cardiomyocytes, the stimulation of 5-HT_4_-receptors exerts an inhibition of the gap junction current, while the activation of 5-HT_2_-receptors increases the gap junction current [166].

In patients with chronic atrial fibrillation, the mRNA expression of the 5-HT_4_-receptors increased significantly [213]. However, in our opinion, this increase is not splice-variant-specific because the mean values of the mRNA expression of 5-HT_4a_- and 5-HT_4c_-receptors were also higher than in the control samples from patients with sinus rates. However, there was high scatter in the data, and more experiments are needed. Moreover, in chronic atrial fibrillation, 5-HT_4g_-receptors were hardly detectable. Significant is the hint from their data that these increases might be a compensatory mechanism that perhaps spiralled out of control. In samples from patients in the initial stages of atrial fibrillation (acute fibrillation), the expression of 5-HT_b_-, 5-HT_c_-, and 5-HT_g_-receptors had lower mean values than in sinus rhythm control samples [213]. Others reported that in patients with atrial fibrillation lasting over one year, the stimulatory function of serotonin in the pacemaker I_f_-current was not different from in patients in sinus mode, suggesting that this pathway is unaltered in supraventricular arrhythmias in humans [159].

### 17.4. Ischaemia and Hypoxia

When cardiac ischaemia was stimulated in isolated perfused hearts, 5-HT_4_-TG exhibited a faster contractile decline in the force of contraction after initiating no-flow ischaemia [225]. This might indicate a detrimental role of 5-HT_4_-receptors in cardiac ischaemia. After experimental hypoxia, atrial preparations from 5-HT_4_-TG increased in the force of contraction faster than in WT [225]. This might indicate that the 5-HT_4_-receptor has a cardiac protective role against hypoxic injuries. Moreover, 5-HT_4_-receptors reduced the uptake of Ca^2+^ into mouse mitochondria under normoxic conditions, but increased this uptake under hypoxic conditions, confirming the role of 5-HT_4_-receptors in cardiac hypoxia [163].

The longstanding literature shows that serotonin can also cause ischaemia by the direct vasoconstriction of coronary arteries in experimental animals via altered 5-HT_2A_-receptors [250]. On a molecular basis, the 5-HT_1b_-receptor after stimulation with 5-HT shows altered epigenetic response in covalent modifications of nuclear proteins in cells similar to pulmonary vascular cells from patients with pulmonary hypertension, supporting a clinically relevant role of 5-HT in pulmonary hypertension at least [251].

### 17.5. Sepsis

In a study on isolated perfused rat hearts from rats with caecal-lesion-induced sepsis or control-treated rats, the authors observed that 5-HT prolonged the atrial action potential, increased heart rate, and aggravated myocardial injury [252]. They suggested that serotonin might therefore lead to cardiac dysfunction in sepsis [252]. However, these interesting data are difficult to translate into the clinic, because rat hearts usually express 5-HT_2A_-receptors, and also manifested 5-HT_4_-receptors in heart failure. Human hearts express no inotropically active 5-HT_2a_-receptors [59].

In LPS-induced sepsis in rats, increased vasodilation and a reduced response to 5-HT-mediated vasoconstriction were noted. This was accompanied and tentatively explained by the increased mRNA expression of 5HT_1A_-receptors and reduced mRNA expression of 5HT_1B_-, 5HT_1D_-, and 5HT_2A_-receptors in septic aortae compared with control tissue [253].

Interestingly, in a recent observational study on septic patients in France, sepsis was not accompanied by an alternation in plasma levels of serotonin, but with a profound reduction in serotonin levels in platelets (627 vs. 222 nM), suggesting a role of serotonin in human sepsis, as recently reviewed [254]. Serotonin in platelets has recently been suggested to contribute to shock in mouse models [255]. In a mouse model of caecal ligation and puncture (CLP-induced sepsis), the WT group exhibited higher mortality than the TPH1-KO mice (with a lower level of peripheral serotonin). This suggests to the authors that there might be a detrimental effect of serotonin, at least in this model of sepsis [256].

### 17.6. Carcinoid Syndrome

Malignant carcinoid tumours have been known since at least 1890 (review: [257,258], and are well known to have detrimental effects on cardiac function [259,260,261,262]. Finally, heart failure can be a long-term consequence of carcinoid syndrome [54,263,264]. Carcinoid syndrome typically arises in tumours originating in enterochromaffin cells of the gut. However, carcinoid tumours can also follow from primary tumours located in the ovaries [212] and pancreas [247]. Carcinoid syndrome can manifest as atrial fibrillation [265] or left- or right-sided heart failure [266].

### 17.7. Hypothermia and Hyperthermia

It has long been known that temperature changes can alter the basal force of contraction in the human heart. There are regional differences. In the human ventricle, an increase in temperature reduces the force of contraction, whereas a decrease in temperature increases the force of contraction (e.g., [267]). In the human atrium, the opposite holds true; an increase in temperature is accompanied by an augmentation of the force of contraction, and in the human atrium, hypothermia reduces the force of contraction. Their differences are usually explained by the opposite regulation of Ca^2+^ levels in these regions [111]. Interestingly, the basal beating rate was lower in hypothermia [111], and the positive chronotropic effect of 5-HT in 5-HT_4_-TG was attenuated in hypothermia compared with normothermia [111]. This might mean, under clinical conditions (such as the use of artificial hypothermia in surgery), that the contractile, and thus, functional, response of endogenous 5-HT to increase the beating rate and force of contraction is expected to be blunted. However, this has never been directly studied in the human heart, in vitro or in vivo, and might be a new research aspect.

### 17.8. Ageing

In ageing, 5-HT uptake into platelets is increased. Therefore, 5-HT concentrations in the platelets are higher, and 5-HT is more likely to induce platelet aggregation [268,269,270,271]. As mentioned elsewhere, the expression, and thus, the function of the 5-HT_4_-receptor is developmentally regulated, at least in the rat heart; some indirect evidence points to a similar situation in the human heart. In the rat foetus, the 5-HT_4_-receptor is functional and highly expressed; this expression is primarily lost after birth. However, re-expression and regained function occur with cardiac pathologies (heart failure) in the rat and the human heart.

In pigs, the positive inotropic effect of 5-HT in the atrium and ventricle in vitro is weaker in neonates than in adulthood [84,112]. The potency to increase the force of contraction was approximately 15 times higher (0.060 versus 0.89 µM), and the efficacy nearly doubled in isolated muscle strips from three-month-old pigs compared with neonatal pigs [84]. Likewise, the increase in the PKA activity by the application of 5-HT in these muscle strips was higher in older than younger pigs [84]. The opposite occurs in rats; foetal rat hearts have high levels of 5-HT_4_-receptors, which goes hand in hand with a significant positive inotropic effect of 5-HT in neonatal cardiac preparations. Remarkably, the expression of 5-HT_2A_- and 5-HT_2B_-receptors markedly increased in foetal rat hearts and peaked at birth, but fell after that [130]. This was explained by the physiological shift from maternal to neonatal circulation; this will increase the pressure felt by the left ventricle [130]. In contrast, in adult rats, 5-HT is devoid of a positive inotropic effect in the rat ventricle [80,130]. In human atria, 5-HT activates AC less in aged individuals, which might be caused by increased levels of Gi proteins in these hearts [98]. Upon ageing, the activity of MAO-A increases in the rat heart, potentially leading to the faster degradation of 5-HT to toxic products, cardiac dysfunction and arteriosclerosis [272].

In mouse and rat hearts, the expression of the 5-HT_4_-receptors is high in the foetal atrium and ventricle and is hardly detectable after birth in mice and rats [166,176]. In rats, this expression of the 5-HT_4_-receptor correlated with function; in the foetal rat heart, 5-HT acts via 5-HT_4_-receptors. In healthy adult rat hearts, 5-HT does not act via 5-HT_4_-receptors [80,166]. Similar data in foetal human hearts do not seem to be available, but might interest neonatologists.

## 18. Possible Cardiac Side Effects of Serotoninergic Drugs

1. One can write a long list of clinically used drugs known to increase serotonin levels in plasma. In this context, one can start with drugs that inhibit the degradation of serotonin. Examples are tranylcypromine and moclobemide. Both drugs are used as a second choice if one must treat a patient with depression. Moclobemide acts primarily by inhibiting the activity of MAO-A, whereas tranylcypromine inhibits both MAO-A and MAO-B. Thus, both drugs should increase levels of 5-HT in the brain, which is the intended effect. However, there is no reason why they should not also elevate cardiac 5-HT levels. We have presented evidence that both can first elevate cardiac 5-HT concentrations and thereby stimulate 5-HT_4_-receptors in the heart and potentiate the inotropic effect of 5-HT on the force of contraction [29].
2. SERT inhibitors also elevate local concentrations of 5-HT. The classical example is the unspecific inhibitor of monoamine uptake: cocaine. Cocaine is known to potentiate the cardiac function of 5-HT via 5-HT_4_-receptors in the human heart [59]. This can lead to cardiac arrhythmias [59]. This side effect of cocaine is, in part, explained by its ability to inhibit the transport of 5-HT via SERT. However, to treat depression, many SERT-selective (e.g., sertraline, fluoxetine, and citalopram) or unselective reuptake inhibitors (e.g., venlafaxine) are known and are expected to act at least qualitatively like cocaine in the human heart. At least for citalopram, drug regulating agencies have made warning advice mandatory because of arrhythmia propensity [273].
3. If the production of 5-HT increases, then higher levels in the heart of 5-HT are predictable. The precursor of 5-HT, called 5-HTP (Figure 1), has been advocated as an add-on to treating depression because 5-HTP is metabolised in the human body to 5-HT. However, 5-HTP after conversion to 5-HT in the heart or other organs, has also been known to activate 5-HT_4_-receptors in the human heart [29], and thus might lead to arrhythmias.
4. More recent data from our lab indicate that the active principles of magic mushrooms, namely, psilocin and psilocybin, also stimulate human cardiac 5-HT_4_-receptors [274]. Psilocybin has recently been approved in the USA to treat some forms of depression [275].
5. Similarly, hallucinogenic drugs from the Amazon basin, currently tested in humans for various psychiatric diseases such as 5-methoxy-dimethyltryptamine, not only stimulate pig cardiac 5-HT_4_-receptors [118], but also human cardiac 5-HT_4_-receptors [276].
6. A venom from frogs, called bufotenine, an isomeric form of psilocin, not only stimulates pig cardiac 5-HT_4_-receptors [118], but also activates 5-HT_4_-receptors in the human heart. Thus, it may lead to arrhythmias [277].
7. Ergotamine and LSD stimulate 5-HT_1B_-receptors directly but also detrimental 5-HT_2B_-receptors, leading to valve fibrosis. Recent laboratory data indicate that LSD and ergotamine can also directly activate human 5-HT_4_-receptors in the heart, and thus may lead to arrhythmias [278].
8. 5-HT_4_-receptor agonists (full and partial agonists) are now prescribed to treat irritable bowel disease (e.g., metoclopramide, prucalopride, [279]), an overactive urine bladder [280], and Alzheimer’s disease (e.g., [281]). The indication of irritable bowel syndrome for these drugs has been questioned; the side effects might be more severe than the disease [282]. Interestingly, metoclopramide (known to stimulate 5-HT_4_-receptors in the gut and block 5-HT_3_- and D_2_-receptors in the brain) acts as an agonist in atrial preparations from human hearts [245]. When metoclopramide was studied in more detail in 5-HT_4_-TG, it turned out to be a partial agonist concerning inotropy in left atrial preparations of 5-HT_4_-TG. It was ineffective in the WT preparations. Moreover, metoclopramide increased the left ventricular ejection fraction in anaesthetised 5-HT_4_-TG, but not in WT. This suggests that metoclopramide can also stimulate ventricular 5-HT_4_-receptors in principle. Moreover, metoclopramide can be stimulated as a partial agonist 5-HT_4_-receptor in the isolated human atrium [238]. However, whether metoclopramide acts as an agonist on the human ventricle, and thereby increases the force of contraction, has not yet been studied. However, it would lend more clinical relevance to these studies [238]. Some other gastrointestinal drugs are agonists at the 5-HT_4_-receptor. These include cisapride, renzapride, zacopride, and tegaserod [59,83,89,103,118,162,182,283,284,285,286,287]. These agonists were intended to treat hyperactive bowel movements. However, these drugs can be absorbed and reach the heart, stimulating cardiac 5-HT_4_-receptors and leading to tachycardia and other arrhythmias. Zacopride, similarly to tropisetron, also blocks 5-HT_3_-receptors; in animal models, it is active as an antiemetic drug [288]. Somewhat surprisingly, at least in transfected HEK cells, artificial agonists such as prucalopride or cisapride were much more effective than 5-HT in increasing Ca^2+^ transients [216]. This result contradicts the contraction data in animal preparations expressing 5-HT_4_-receptors or human atrial preparations, suggesting differences in signal transduction. In human atrial myocytes, prucalopride concentration dependently increased I_Ca.L_ with a maximum response at 10 µmol/L. However, this effect was lower than that reached with 5-HT. Moreover, 5-HT applied subsequently to the maximum concentration of prucalopride further enhanced I_Ca.L_, indicating that prucalopride acts as a partial agonist on I_Ca.L_ in these cells [101]. Additionally, the finding that cisapride and prucalopride could not elevate calcium transients in 5-HT_4b_-receptor-transfected cells is puzzling [216]. In contrast, cisapride elevates the force in human atrial preparations [89], where the 5-HT_4b_-receptor is highly expressed, and thus should carry most of the contractile response of the agonist. It may be relevant in this context that, at least in transfected HEK cells, cisapride was more effective in stimulating cAMP levels via 5-HT_4b_-receptors, whereas prucalopride was more potent compared with 5-HT_4a_-receptors [216]. Cisapride and renzapride are partial agonists at low 5-HT_4_-receptor levels and full agonists at high 5-HT_4_-receptor levels [182]. Mosapride inhibited the proliferative activity of human umbilical-vein-derived cultured endothelial cells (HUVECs), dependent on cell cycle arrest and not apoptosis [289]. Prucalopride is a partial agonist in human atrial and left or right neonatal pig atrial preparations via 5-HT_4_-receptors [290]. 5-HT was less potent than prucalopride, but equally effective [162]. 5-HT_4_-receptor agonists have been suggested in treatments for sinus bradycardia and to accelerate atrioventricular conduction in the heart [291]. However, clinical studies in this regard are currently lacking. At least one clinical study has reported arrhythmias in healthy volunteers [292] taking prucalopride. This view is supported by animal studies. At least in atrial preparations from 5-HT_4_-TG, prucalopride induced a concentration-dependent positive chronotropic effect. There is evidence that prucalopride and cisapride stimulate atrial and ventricular 5-HT_4_-receptors under these conditions [162].
9. In addition, there are novel dually active experimental drugs to treat Morbus Alzheimer’s that are intended to stimulate 5-HT_4_-receptors in the brain, but can also stimulate 5-HT_4_-receptors in the heart, an unintended side effect of these drugs. The 5-HT_4_-receptor agonist (RS 67333) donecopride might be useful in treating Morbus Alzheimer [293]. However, this compound could induce arrhythmias because it is expected to not only stimulate the brain, but also cardiac 5-HT_4_-receptors, which has not yet been reported.

## 19. Receptor Antagonists Available and Their Putative Therapeutic Use

Tropisetron blocks 5-HT_3_-receptors, and therefore was used as one of the first drugs clinically against 5-HT-induced emesis (e.g., in cancer therapy). Moreover, tropisetron can also block 5-HT_4_-receptors. In this way, tropisetron can antagonise the positive inotropic effects of 5-HT_4_-receptor stimulation in the human heart [29]. We have suggested that arrhythmias caused by agonists might be treated in patients with the approved drug tropisetron [110]. Piboserod is antagonistic at 5-HT_4_-receptors [224] and has been attempted with some success in patients. The authors used piboserod to reduce the development of cardiac hypertrophy in heart failure patients [224]. Therefore, piboserod might also be tested to treat patients with atrial fibrillation, the most common arrhythmia. Indeed, 5-HT_4_-receptor antagonists have been successfully used in animal studies and some patient studies for supraventricular arrhythmias [96,247], but did not enter the clinic due to side effects (RS-100302: [121]).

Nevertheless, further work using antagonists at the 5-HT_4_-receptor to treat heart failure and arrhythmias is meaningful and should be encouraged. Interestingly, an endogenous antagonist may exist in human cardiac 5-HT4-receptors. L-lysine, the amino acid, antagonised positive inotropic effects in left atrial preparations of 5-HT4-TG [294,295]. The physiological relevance is unclear. We speculated that L-lysine might be an endogenous antiarrhythmic agent that could be tested in a clinical trial to suppress atrial fibrillation [294].

## 20. Summary

In this study, we reviewed the cardiac role of serotonin. Serotonin is produced in peripheral tissue, including the heart. Serotonin acts on several serotonin receptors in the heart. In the human heart, the inotropic and chronotropic, but also proarrhythmic effects of serotonin are mediated by 5-HT_4_-receptor isoforms. The expression of 5-HT receptors might be altered in cardiovascular disease. The signal transduction of the 5-HT_4_-receptors is only starting to unravel. A better understanding might lead to novel transduction-specific agonists and antagonists. Selective agonists or antagonists at 5-HT_4_-receptors are currently not used to treat cardiac diseases. Therefore, it might make sense to re-evaluate 5-HT_4_-receptor agonists and antagonists in cardiovascular diseases.

## Figures and Tables

**Table 1:** Synopsis of 5-HT receptor function in the hearts of several species. We differentiate species, the region or tissue in this species, the agonists and antagonists tested in that region, the mechanical response (positive inotropic effect: PIE), positive chronotropic (PCE), no measurable response (NR), biochemical measurements for signal transduction of the 5-HT_4_-receptor, in human samples with underlying disease (coronary heart disease (CAD), heart failure (HF), normorhythmia (SR), chronic atrial fibrillation (CAF), paroxysmal atrial fibrillation (PEF), Mice with cardiac specific overexpression of the 5-HT_4_-receptor (5-HT_4_-TG) or wild type littermates (WT); No: no effect; PIE: positive inotropic effect, PCE: positive chronotropic effect; cAMP: increase in the heart content of cAMP (3′,5′-cyclic adenosine monophosphate); PKA: increase in the activity ratio of cAMP dependent protein kinase. If: increase in current through funny channel. LA: isolated left atrium; RA: isolated right atrium; LTCC: current through the L-type calcium ion channels in the appropriate tissue was increased by 5-HT. Tropisetron was initially called SB205930. AC: cAMP producing activity of adenylate cyclase was found enhanced. Ve: ventricle, usually papillary muscles were studied. PDE-I: in the presence of a phosphodiesterase inhibitor, usually isobutylmethylxanthine. PLB-P: increase in phosphorylation state of phospholamban, TnI-P: increase in the phosphorylation state of the inhibitory subunit of troponin. Superscripted number in the last column are correspondent with numbers in previous columns, indicate for instance in which citation this drug was used or this enzyme was studied.

Number	Species, Region	Receptor for Inotropy	Agonist	Antagonist	Biochemistry	Disease	Mechanical Function	Reference
#1	human ventricle	5-HT_4_	5-HT	^1^GR113808	^2,4^LTCC: no ^5^AC: no		^1^PIE always in the presence of PDE-I In the absence of PDE-I: ^2,3^no effect, ^1^sometimes PIE	^1^Brattelid et al., 2004 [84] ^2^Jahnel, 1992 [2] ^3^Schoemaker et al., 1993 [82] ^4^Ouadid et al., 1995 [85] ^5^Brodde et al., 1998 [86]
#2	human left atrium	5-HT_4_	5-HT	tropisetron (ICS205930)	cAMP PKA	Terminal heart failure	PIE	Sanders and Kaumann, 1992 [87]
#3	human right atrium	5-HT_4_	^8,25,28^5-HT ^8,28^5-MeOT ^8^renzapride ^8^BIMU8 ^29^zacopride ^23,24,28^prucalopride ^27^metoclopramide ^28^tegaserod ^28,29^cisapride	^1,2,15,16,30^tropisetron (ICS205930) ^16^SDZ205557 ^17,22,24^GR113808 ^18^SB207710 ^13,19,25^SB203186 ^11^DAU6285 ^11,25^GR125487 ^25^GR113808	^1,2,5,6,10^cAMP ^1,2^PKA ^4,8,9,20,22,23^LTCC ^11,12^If ^16,21,22^AC ^25^PLB-P ^25^TnI-P	Coronary heart disease, terminal heart failure, mitral valve disease, aortic valve disease	^1,15,2,4,7,10,14,16, 17,18,19,22^PIE Atrial isolated cardiomyocytes: ^10^PIE	^1,2^Kaumann et al., 1990 [59] ^3,9^Jahnel et al., 1993 [88] ^4^Jahnel et al., 1992 [81] ^5^Kaumann et al., 1990 [59] ^6,7^Kaumann et al., 1991 [89] ^8^Ouadid et al., 1992 [90] ^10^Sanders et al., 1995 [91] ^11^Pino et al., 1998 [92] ^12^Workman and Rankin, 1998 [26] ^13^Parker et al., 1995 [93] ^14^Zaizen et al., 1996 [94] ^15^Schoemaker et al., 1993 [82] ^16^Zerkowski et al., 1993 [95] ^17^Kaumann, 1993 [96] ^18^Kaumann et al., 1994 [97] ^19^Kaumann and Sanders 1994 [27] ^20^Ouadid et al., 1995 [85] ^21^Brodde et al., 1995 [98] ^22^Pau et al., 2003 [99] ^23^Pau et al., 2007 [100] ^24^Pau et al., 2005 [101] ^25^Gergs et al., 2009 [70] ^26^Gergs et al., 2017 [29] ^27^Neumann et al., 2021 [102] ^28^Chai et al., 2012 [83] ^29^Conlon et al., 2018 [103]
#4	human right atrium	5-HT_4_	^1,2^5-HT		^2^LTCC ↓ ^2^PLB-P↓ ^3^cAMP↓	^1^Effect of 5-HT on LTCC ↓	^2,3^PIE ↓	^1^Pau et al., 2007 [100] ^2^Christ et al., 2014 [104] ^3^Berk et al., 2016 [105] ^2^Gergs et al. [106]
#5								
#6	human sinus node	5-HT_4_					^1,2^in vivo: PCE ^3^in vitro	^1^Hollander et al., 1957 [107] ^2^Le Messurier et al., 1959 [108]
#7	human coronary artery	5-HT_1D_						
#8	mouse ventricle	WT (no) 5-HT_4_-TG	^1,2,3,4^5-HT ^5^metoclopramide ^4^cisapride ^4^prucalopride	^1^GR125487		^1,5^PLB-P 1TnI-P	^1,2,3,4,5^PIE ^1,2,4,5^PCE	^1^Gergs et al., 2010 [40] ^2^Gergs et al., 2017 [29] ^3^Gergs et al., 2017 [109] ^5^Neumann et al., 2021 [102]
#9	mouse left atrium	WT (no) 5-HT_4_-TG	^1,2,5,6^5-HT ^6^metoclopramide ^4^cisapride ^4^prucalopride	^1,3^GR125487			^1,2,3,4,5,6,7^PIE	^1^Gergs et al., 2010 [40] ^2^Gergs et al., 2017 [29] ^3^Gergs et al., 2017 [109] ^5^Neumann et al., 2019 [110] ^6^Neumann et al., 2021 [102] ^7^Gergs et al., 2021 [111]
#10	mouse right atrium	WT (no) 5-HT_4_-TG	^1^5-HT ^5^metoclopramide ^4^cisapride ^4^prucalopride		^1^GR125487		^1,2,4,5,6^PCE	^1^Gergs et al., 2010 [40] ^2^Gergs et al., 2017 [109] ^4^Neumann et al., 2019 [110] ^5^Neumann et al., 2021 [102] ^6^Gergs et al., 2021 [111]
#11	porcine ventricle	5-HT_4_	5-HT	^1^SB207710 ^1^GR113808	^1^PKA		PIE only with PDE-I	^1^Brattelid et al., 2004 [84] ^2^De Maeyer et al., 2006 [112] ^3^Schoemaker et al., 1992 [113]
#12	porcine left atrium	5-HT_4_	^1,2,3^5-HT ^2,3,4,5^prucalopride ^2^R149402 ^2^R199715 ^2^tegaserod ^5^cisapride	^2^GR113308		^3^PLB-P ^3^TnI-P	^2,3,4^PIE increased with of PDE-I	^1^Parker et al., 1995 [93] ^2^De Maeyer et al., 2006 [112] ^3^Weninger et al., 2013 [114] ^4^Weniger et al., 2014 [115] ^1^Kaumann et al., 1995 [116] ^5^Conlon et al., 2018 [103]
#13	porcine right atrium	^9^5-HT_4_	^2,5,7,8^5-HT ^2^cisapride ^2^renzapride ^2^5-carboxam-idotryptamine ^5,8^prucalopride ^5^R149402 ^5^R199715 ^5^tegaserod	^2,9^tropisetron (ICS 205930) ^5^GR113308		^7^cAMP	^1,2,3,4,5,6,7^PCE	^1^Bom et al., 1988 [117] ^2^Kaumann, 1990 [96] ^3^Kaumann, 1994 [28] ^4^Medhurst and Kaumann, 1993 [118] ^5^De Maeyer et al., 2006 [112] ^6^Parker et al., 1995 [93] ^7^Galindo-Tovar et al., 2009 [119] ^8^Conlon et al., 2018 [103] ^9^Schoemaker et al., 1992 [113]
#14	Anaestheti-sed pig	5-HT_4_	^1,2^5-HT ^1^5-Methoxytryptamine ^1^renzapride ^5^prucalopride	^2,3^tropisetron ^1^ICS205-930 ^4^RS-100302			^1,2,3^PCE	^1^Villalón et al., 1991 [120] ^2^Parker et al., 1995 [93] ^3^Bom et al., 1988 [117] ^4^Rahme et al., 1999 [121] ^5^Conlon et al., 2018 [103]
#15	cat	^3^LA: 5-HT_3_ ^3^Ve: 5-HT_4_	^3^5-HT	^3^LA: methysergide ^3^Ve: phenoxybenzamine			^1^PCE ^2,3,4^LA: Ve: PIE	^1^Saxena et al., 1985 [122] ^3^Kaumann, 1985 [123] ^4^Kaumann et al., 1990 [59] ^5^Buccino et al., 1967 [76]
#16	rat	^1^5-HT_2_			^2^no increase in cAMP		^1,3^LA: PIE, ^3,5^No ^1,4^RA:PCE ^1^Ve: no	^1^Läer et al., 1998 [80] ^2^Fischer et al., 1995 [124] ^3^Ouadid et al., 1992 [90] ^4^Docherty, 1988 [125] ^5^Zaizen et al., 1996 [94]
	rat with aortic banding	5-HT_4_ 5-HT_2A_	5-HT	ketanserin GR113808			Ve: PIE	Brattelid et al., 2007 [126]
	rat with myocardial infarction	^1,2^5-HT_4_ ^2^5-HT_2A_	^1^5-HT ^1^RS67506 ^3^SB207266	^1,2,4^GR113808 ^2,4^ketanserin	Ve: ^1^increase in cAMP also in ^1^cardiomyocytes		^1,2,3^Ve PIE	^1^Qvigstad et al., 2005 [127] ^2^Qvigstad et al., 2005 [128] ^3^Birkeland, 2007 [129] ^4^Brattelid et al., 2012 [130]
#17	guinea pig	^1^5-HT_3_	^1^5-HT ^1^chlorophenyldiguanide	^1^ondansetron	^3,4^release of NA ^1^reserpine-treated		LA: ^1^PIE, ^5^No PIE RA: ^2,3^PCE ^4^Ve: PIE	^1^Tramontana et al., 1993 [131] ^2^Walter et al., 1984 [132] ^3^Trendelenburg, 1960 [79] ^4^Zaizen et al., 1996 [94] ^5^Ouadid et al., 1992 [90]
#18	dog	No			^1^release of NA		^1^LA: PCE, PIE ^1,2^Ve: PIE ^2^In vivo: PIE	^1^Chiba, 1977 [133] ^2^Buccino et al., 1967 [76]
#19	rabbit	No			^1,2^release of NA		LA: ^1^PIE ^3^No PIE ^2^Whole Heart	^1^Trendelenburg, 1960 [79] ^2^Fozard and Mwaluko, 1976 [134] ^3^Ouadid et al., 1992 [90]
#20	frog	No					Atrium: ^1,2^No PIE	^1^Ouadid et al., 1992 [90] ^2^Hanson and Magill, 1962 [135]
#21	molluscs	No	5-HT	^3^lisuride ^4^methysergide	^3,4^cAMP in auricles and ventricles		^1,2,4^PCE ^1,2,3,4^PIE	^1^Greenberg, 1960 [136] ^2^Erspamer and Ghiretti, 1951 [137] ^3^Kebabian et al., 1979 [138] ^4^Sawada et al., 1984 [139]
#22	turtle	No					No effect	Hanson and Magill, 1962 [135]
#23	monkey	^1^No ^2^5-HT_4_			^1^release of NA	^2^mRNA of 5-HT4-receptor detected	^1^Indirect PIE	^1^Zaizen et al., 1996 [94] ^2^Mader et al., 2006 [140]

## Data Availability

Data available on reasonable requests addressed to the senior author.
